# A new genus for the western Caucasian *Lyurella* (Amphipoda, Crangonyctidae), with descriptions of three new hypogean species from the western foothills of the Russian Caucasus

**DOI:** 10.3897/zookeys.1294.206924

**Published:** 2026-10-13

**Authors:** Ivan N. Marin, Dmitry M. Palatov

**Affiliations:** 1 A.N. Severtsov Institute of Ecology and Evolution of RAS, Moscow 119071, Russia A.N. Severtsov Institute of Ecology and Evolution of RAS Moscow Russia

**Keywords:** Barcoding, Crustacea, diversity, historical reconstruction, Palearctic, phylogeny

## Abstract

A new genus, *Caucasirella***gen. nov**. (Crustacea, Amphipoda, Crangonyctidae), is proposed for the Caucasian representatives of the genus *Lyurella* Derzhavin, 1939, namely *Caucasirella fontinalis* (Marin & Palatov, 2021), **comb. nov**. (the type species), *Caucasirella shepsiensis* (Sidorov, 2015), **comb. nov**., *Caucasirella asheensis* (Palatov & Marin, 2021), **comb. nov**., *Caucasirella fanagorica* (Palatov & Marin, 2021), **comb. nov**., and *Caucasirella mikhailovi* (Marin & Palatov, 2021), **comb. nov**. Three new species, *Caucasirella shapsuhica***sp. nov**., *Caucasirella mzymtiensis***sp. nov**., *Caucasirella lazareviensis***sp. nov**., are also described from the hypogean (riverine hyporhea, springs) habitats of western foothills of the Russian Caucasus. An integrative approach using uni-locus (COI) phylogeny, morphological and geographical data clearly support the creation of a new genus for Caucasian species. *Caucasirella***gen. nov**. could be separated from Hyrcanian *Lyurella hyrcana* Derzhavin, 1939 mainly by the presence of two or three coupling hooks in the retinacules of pleopods (vs. four hooks), a deep distal notch on the telson and, most notably, the absence of a peduncular distoventral distal serrate process of the uropod I in ♂♂. They probably diverged ca 10–7.5 Mya (Miocene), during the uplift of the area of the Caucasian Ridge. The morphology of juveniles of *Caucasirella lazareviensis***sp. nov**., described for the first time, revealed their significant difference from adults and important morphological features for the entire “*Synurella*” clade.

## Introduction

The crangonyctid amphipod genus *Lyurella* Derzhavin, 1939 (Crustacea, Amphipoda, Crangonyctidae) currently encompasses two geographically distinct groups of species: *L. hyrcana* Derzhavin, 1939 (the type species of the genus) (+ several possibly undescribed species) is known from the southeastern and southern Caspian coastal habitats of Azerbaijan and Iran (the Hyrcanian refuge) (Derzhavin 1939; Ruffo 1974; Aliev 2000), while several species (see below) also referred to the genus *Lyurella* have been described from the Russian part of the northeastern Black Sea coastal habitats (mostly the Colchis refuge) (Sidorov 2015; Marin and Palatov 2021a). Molecular genetic analysis has established the phylogenetic position of the genus within the “*Synurella*” clade of the family Crangonyctidae, also including such genera as *Synurella* Wrześniowski, 1877, *Eosynurella* Martynov, 1931, and *Palearcticarellus* Palatov & Marin, 2020 (Copilaş-Ciocianu et al. 2019; Palatov and Marin 2020; Marin and Palatov 2021a, 2021b, 2023a, 2023b, 2025; Copilaş-Ciocianu et al. 2024). Within the clade, *Lyurella* is considered as a sister taxon to the genera *Synurella* Wrześniowski, 1877 (Marin and Palatov 2021b, 2025) or *Palearcticarellus* Palatov & Marin, 2020 (Marin and Palatov 2021a; Copilaş-Ciocianu et al. 2024). Morphologically, the genus can be distinguished from related genera in the clade by its relatively small size, the presence of additional spines on the ventral (inner) margin of the dactyli of the ambulatory pereopod, completely fused urosomites, and a vestigial squamiform uropod III with completely reduced distal article.

Until the present time, five species of the genus *Lyurella*, namely *L. shepsiensis* Sidorov, 2015, *L. fontinalis* Marin & Palatov, 2021, *L. asheensis* Palatov & Marin, 2021, *L. fanagorica* Palatov & Marin, 2021, and *L. mikhailovi* Marin & Palatov, 2021, have been described from the hyporheic habitats of the mountainous rivers of the southwestern foothills of the Greater Caucasian Ridge (Sidorov 2015; Marin and Palatov 2021b). The interspecific genetic divergence based on the COI mtDNA gene marker, among the Caucasian species of the genus studied, ranged from 11% to 21%, indicating a long-term period of isolation and lack of gene flow, at least since the Pliocene (Marin and Palatov 2021b). The lowest level of genetic divergence between the described species, ~ 4%, was considered to be species-specific due to the presence of distinct morphological differences. Meanwhile, the richness of these crangonyctid amphipods remains underexplored, particularly in the western foothills of the Great Caucasus.

During the ongoing zoological research in 2023–2025, new locations and habitats of *Lyurella* were discovered in the western foothills of the Russian Caucasus. Through a comprehensive approach to studying the morphology and genetics of the collected specimens, it was revealed that they belong to distinct species; even at the generic level, they could be separated from *L. hyrcana*. In this article, we describe three newly discovered crangonyctid amphipod species, collected from various hypogean habitats, significantly expanding the known range of these interesting crangonyctid amphipods and their distribution in mountainous areas of the northwestern Caucasus. Moreover, a new genus is proposed for the western Caucasian representatives of the genus *Lyurella* Derzhavin, 1939.

## Materials and methods

### Sample collection and processing

Amphipods were collected using a hand net various subterranean and epigean water resources of the western foothills of the Great Caucasian Ridge (see below) in 2023–2025. After sampling, they were preserved in 90% solution of ethanol.

### Material deposition

The type material was deposited in the collection of the Zoological Museum of Moscow State University, Moscow (**ZMMU**). Additional material was deposited in the authors’ personal collection in A.N. Severtsov Institute of Ecology and Evolution of Russian Academy of Sciences, Moscow, Russia (**LEMMI**).

### Morphological study

Living amphipods were photographed using a Canon GI6 digital camera. Fixed individuals were dissected using a Lomo MBS-10 light binocular microscope, tweezers, and dissecting needles. The prepared appendages and body parts were placed on slides in glycerin or polyvinyl lactophenol, covered with a cover glass and bordered with transparent nail polish. Slides were photographed with a digital camera attached to an Olympus SX10 light microscope at standard magnifications of ×5, ×7, and ×10. Additional line drawings were made using camera lucida attached to Olympus SZX10 light stereomicroscope. The article presents photographs of only the important distinguishing morphological features of the described species, whereas photographs of all morphological features of closely related species can be found in Marin and Palatov (2021).

Scanning electron microscope (SEM) images were made at the Paleontological Museum of the Paleontological Institute of the Russian Academy of Sci­ences, Moscow, using Vega3 Tescan microscope. The samples placed in 95% ethanol were cleaned in an ultrasonic cleaner, followed by dehydration with acetone and critical-point drying (CPD). Subsequently, they were affixed to specimen stubs with double-sided tape, and finally coated with gold through sputtering using the Polaron PS 100.

Body length (bl., mm) was measured as the dorsal length from the distal margin of the head to the posterior margin of the telson, with the exclusion of both antennae and uropod III.

### Amplification and sequencing of DNA

The mitochondrial cytochrome c oxidase subunit I (COI mtDNA) has proved extremely informative in previous studies at both population and species levels (Avise 1993; Palatov and Marin 2020; Marin and Palatov 2021a). Total genomic DNA was extracted from muscle tissue using the innuPREP DNA Micro Kit (AnalitikJena, Germany). The COI mtDNA gene marker was amplified with the using of the universal primers LCO1490 (5'–GGTCAACAAATCATAAAGATATTGG–3') and HC02198 (5'–TAAACTTCAGGGTGACCAAAAAATCA–3') (Folmer et al. 1994). Polymerase chain reaction (PCR) was performed on an amplificator T100 (Bio-Rad, USA) under the following conditions: initial denaturation at 96 °C for 1.5 min followed by 42 cycles of 95 °C for 2 min, 49 °C for 35 seconds, and 72 °C for 1.5 min. Each chain reaction was concluded by an extension step, set at 72 °C for 7 min. The volume of 10 µL of reaction mixture contained 1 µL of total DNA, 2 µL of 5 × PCR mix (Dialat, Russia), 1 µL of each primer and 5 µL of H_2_O. The amplification products were separated by using gel electrophoresis of nucleic acids on a 1.5% agarose gel in 1 × TBE, and then stained and visualized with 0.003% EtBr, using imaging UV hardware. PCR products were then sequenced using Genetic Analyzer ABI 3500 (Applied Biosystems, USA) and BigDye 3.1 (Applied Biosystems, USA) with forward and reverse primers. The dataset of aligned sequences of COI mtDNA gene markers, ~ 586 base pairs in length, used in the study were taken from GenBank (NCBI) (Table 1) and generated from this study.

**Table 1.** Pairwise genetic (COI mtDNA) distances (*p*-distance) (substitutions per 100 nucleotides ± SE) between the genera within the “*Synurella*” clade of the family Crangonyctidae.

	***Caucasirella* gen. nov**.	*** Palearcticarellus ***	***Lyurella hyrcana***	*** Eosynurella ***
* Palearcticarellus *	0.256 ± 0.044			
*Lyurella hyrcana*	0.260 ± 0.047	0.259 ± 0.045		
* Eosynurella *	0.276 ± 0.048	0.267 ± 0.046	0.255 ± 0.045	
* Synurella *	0.278 ± 0.047	0.302 ± 0.052	0.303 ± 0.053	0.314 ± 0.056

### Phylogenetic analysis

Obtained sequences were aligned using MEGA 7.0 (Kumar et al. 2016). The best evolutionary substitution model was determined using MEGA 7.0 and jModeltest2.1.141 as GTR+G+I. A phylogenetic analysis was conducted using PhyML 3.0 (http://www.atgc-montpellier.fr/phyml/) (Guindon et al. 2010) with several models based on BIC (Bayesian Information Criterion) and AIC (Akaike Information Criterion). The trees with the higher bootstrap were used for graphic display of relationships within the family and “*Synurella*” clade. Bootstrap support is presented for ML analysis.

Pairwise genetic divergences (*p*-distances) were calculated based on COI sequences using MEGA 7.0 with the Kimura 2‐Parameter (K2P) model of evolution (Kimura 1980).

### Species delimitation

The following three species delimitation approaches were used: distance clustering ABGD (Automatic Barcode Gap Discovery) (Puillandre et al. 2011) and ASAP (Assemble Species by Automatic Partitioning) (Puillandre et al. 2021), phylogeny-aware PTP/bPTP (Poisson Tree Process and its Bayesian variant) (Zhang et al. 2013), and Bayesian GMYC (Generalized Mixed Yule Coalescent) (Pons et al. 2006; Reid and Carstens 2012) as well as morphological evidence.

The ABGD/ASAP analysis was performed using an online version of the program (https://spartexplorer.mnhn.fr/delimitation) with the default program settings.

Poisson Tree Process (PTP) and the Bayesian variant of the method (bPTP) (https://species.h-its.org/) was run on the RAxML gene trees (see above) for 1'10^6^ MCMC (Markov chain Monte Carlo) generations thinning every 1000 and removing the distant outgroup to improve the delimitation results.

The GMYC analysis was run in conjunction with the BEAST2 package (Drummond et al. 2012; Bouckaert et al. 2014, 2019; Drummond and Bouckaert 2015). The MCMC chains were run for 10'10^6^ generations and sampled every 10,000^th^ generations. Bayesian Inference was performed under the GTR model. Following gene tree inference, GMYC was performed using the “splits” R package (SPecies LImits by Threshold Statistics) (Ezard et al. 2009) in R v. 3.5.1 (http://www.r-project.org/) with a single threshold.

Molecular clock analysis was performed based on Bayesian Inference trees performed in the BEAST2 package (Drummond et al. 2012; Bouckaert et al. 2014, 2019; Drummond and Bouckaert 2015) using GTR, TN93, and HKY models, Yule process and Coalescent (constant size) tree priors and strict clock model. The MCMC chains were run for 10^6^ generations sampling every 10^4^ generations were used. The GMYC analysis was implemented as indicated above.

### Time calibration

The estimated minimum (5.16% Mya^-1^) and maximum (as 0.77% Mya^-1^) divergence time were calculated after Guy-Haim et al. (2018), with an average divergence time of 2.5% Mya^-1^ for COI mtDNA gene marker (Lefébure et al. 2006; Copilaş-Ciocianu and Petrusek 2015; Guy-Haim et al. 2018). Additionally, a divergence time estimates as 1.773% Mya^–1^ was calculated according to Copilaş-Ciocianu et al. (2019).

## Results

### Molecular identification

**Figure 1.**
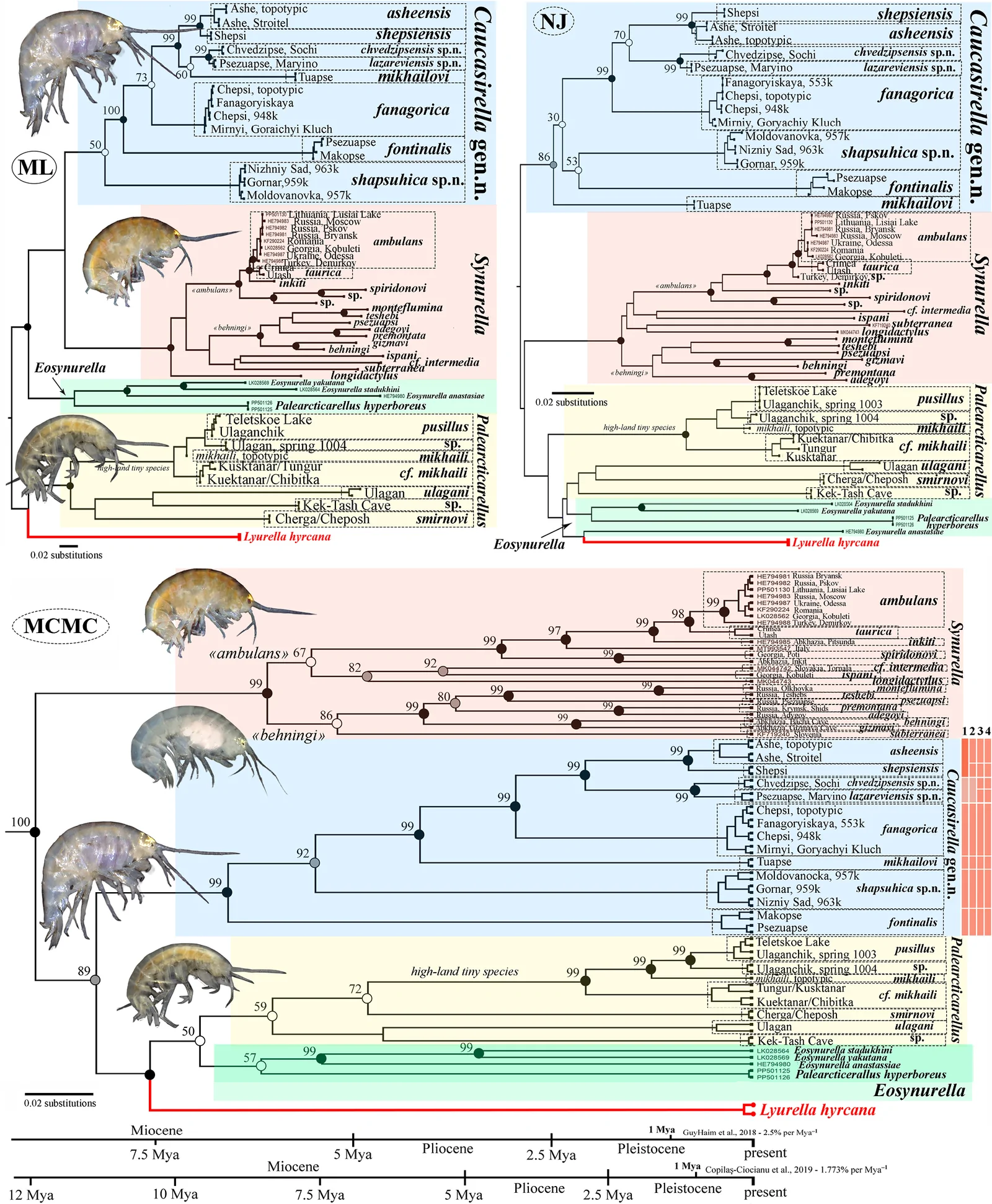
The phylogenetic relationships (ML and NJ trees) and the time-calibrated phylogenetic tree (reconstruction) (MCMC) with the species delimitation analyses based on COI mtDNA gene marker of the studied “*Synurella*” clade of the family Crangonyctidae. Colors indicate different genera; nodes marked with the support based on ML algorithm. Results from different species delimitation methods (1-ABGD; 2-ASAP; 3-PTP/bPTP; 4-morphology) are depicted with colored bars highlighting congruence across methods. The estimated divergence time is indicated after Guy-Haim et al. (2018) and Copilaş-Ciocianu and Petrusek (2015).

The monophyly of the Palearctic “*Synurella*” clade within the family Crangonyctidae, including representatives of the genera *Synurella* Wrześniowski, 1877, *Palearcticarellus* Palatov & Marin, 2020, *Eosynurella* Martynov, 1931, *Lyurella hyrcana* Derzhavin, 1939 (the type species of the genus *Lyurella*) and representatives of *Caucasirella* gen. nov. (Caucasian species previously referring to the genus *Lyurella*) was previously confirmed by several phylogenetic reconstructions (e.g., Palatov and Marin 2020; Marin and Palatov 2021a, 2021b, 2023a, 2023b, 2025; Copilaş-Ciocianu et al. 2024). Our uni-locus (COI-based) phylogenetic reconstructions (Fig. 1) support the isolated position and the monophyly of the Caucasian species of *Caucasirella* gen. nov. (Fig. 1), as well as *L. hyrcana*, which occupies a distant phylogenetic position, with uncertain relationship to the “*Synurella* + *Caucasirella* gen. nov.” clade (see Fig. 1).

The genetic divergence between the different lineages of the generic level within the “*Synurella*” clade of the family Crangonyctidae (see Fig. 1) ranges from 25 to 31% (see Table 1). It is generally comparable to the genetic distance between different genera within other main clades of the family Crangonyctidae (Palatov and Marin 2020; Marin and Palatov 2021a, 2023a, 2023b). The genetic difference between *L. hyrcana* and Caucasian representatives of *Caucasirella* gen. nov. is 0.260 ± 0.047 substitutions per 100 nucleotides (~ 26%), which is comparable to other generic level lineages (genera) within the “*Synurella*” clade (see Table 1).

### Molecular genetic study and species delimitation

The ABGD analysis revealed six operational taxonomic units (OTUs) with a prior interspecific distance of ≤0.0010 (P-value (rank) 0.19–0.20) (combining *C. asheensis* comb. nov. / *C. shepsiensis* comb. nov.; *C. mzymtiensis* sp. nov. / *C. lazareviensis* sp. nov.). The ASAP method identified seven OTUs (combining *C. asheensis* comb. nov. /*C. shepsiensis* comb. nov.), with an interspecies interspecific distance threshold of 0.032809 (3.2%) (P-value (rank) 5.788423e-01), and eight OTUs with an interspecific distance threshold of 0.022674 (2.3%) (P-value (rank) 3.493014e-01). The trees resulting PTP/bPTP method showed similar results with eight OTUs. The GMYC model had a log likelihood of 171.9195, while the null model had a likelihood of 167.8795. As a result of the likelihood ratio test, the null model, which assumes uniform coalescent branching rates throughout the entire tree, was rejected (likelihood ratio = 8.079952, p = 0.01759789*). The number of ML clusters in the analysis was eight (confidence interval 6–9 (95% CI); threshold time: -0.01930606).

The morphological study (see below) also supports the existence of seven separate species, demonstrating clear differences between *C. shepsiensis* comb. nov. and *C. asheensis* comb. nov. (see Palatov and Marin 2021b). Therefore, an integrative approach to species delimitation based on phylogenetic trees (see Fig. 1) supports seven well-separated phylogenetic MOTUs (lineages, species) within *Caucasirella* gen. nov. (see Fig. 1; below).

The interspecific genetic divergence of the COI mtDNA gene marker for the species of *Caucasirella* gen. nov. ranges from 3.3–27.6% (see Table 2). The type species of the genus, *Caucasirella fontinalis* comb. nov. (see below), greatly differs from its congeners for ~ 23–27% (see Fig. 1; Table 2), indicating a long-term isolation. The lowest divergence (~ 3%) was revealed between *C. shepsiensis* comb. nov. /*C. asheensis* comb. nov. and *C. mzymtiensis* sp. nov. / *C. lazareviensis* sp. nov. (see Table 2), which we consider to be separate species (see below). The mean genetic divergence between most species of *Caucasirella* gen. nov. shows a distinct species-specific pattern, with p-distances ranging from 10% to 15% (Table 2).

**Table 2.** Pairwise genetic (COI mtDNA) distances (*p*-distances) (substitutions per 100 nucleotides ± SE) within the studied species of *Caucasirella* gen. nov.

***C. fontinalis* comb. nov. (type species)**		***C. mikhailovi* comb. nov**.	***C. fanagorica* comb. nov**.	***C. shepsiensis* comb. nov**.	***C. asheensis* comb. nov**.	***C. shapsuhica* sp. nov**.	***C. mzymtiensis* sp. nov**.
*C. mikhailovi* comb. nov.	0.276 ± 0.044						
*C. fanagorica* comb. nov.	0.242 ± 0.038	0.157 ± 0.025					
*C. shepsiensis* comb. nov.	0.250 ± 0.039	0.135 ± 0.022	0.118 ± 0.020				
*C. asheensis* comb. nov.	0.243 ± 0.038	0.138 ± 0.022	0.115 ± 0.019	**0.033 ± 0.008**			
*C. shapsuhica* sp. nov.	0.260 ± 0.041	0.176 ± 0.028	0.195 ± 0.031	0.181 ± 0.029	0.182 ± 0.029		
*C. mzymtiensis* sp. nov.	0.251 ± 0.039	0.143 ± 0.024	0.112 ± 0.020	0.091 ± 0.017	0.082 ± 0.015	0.201 ± 0.032	
*C. lazareviensis* sp. nov.	0.233 ± 0.037	0.133 ± 0.022	0.115 ± 0.020	0.084 ± 0.015	0.073 ± 0.014	0.197 ± 0.031	**0.030 ± 0.008**

### Molecular clock analysis

The time of divergence between the generic lineages and the separation of the representatives of *Caucasirella* gen. nov., estimating using *p*-distances (see Materials and methods above) probably occurred during the Miocene, ca 11–8 Mya (min. after Guy-Haim et al. (2018); max. after Copilaş-Ciocianu and Petrusek (2015) (see Fig. 1). The speciation within *Caucasirella* gen. nov. obviously occurred from the late Miocene to the Pleistocene (see Fig. 1), with the “youngest” species (*C. shepsiensis* comb. nov./*C. asheensis* comb. nov. and *C. mzymtiensis* sp. nov./*C. lazareviensis* sp. nov.) diverging in the late Pleistocene, ca 1.2–0.8 Mya (see Fig. 1).

### Taxonomic account

**Order Amphipoda Latreille, 1816**

**Infraorder Gammarida Latreille, 1802**

**Family Crangonyctidae Bousfield, 1973**

#### Caucasirella gen. nov.

Taxon classificationAnimaliaAmphipodaCrangonyctidae

58CB651C-4B3F-5D16-B4CC-26F7C334D275

https://zoobank.org/64E594C3-33E5-4EB9-AF2C-DA623633979F

##### Diagnosis.

**Adults**. Relatively small, total body size varies from 5–7 mm. Body stout, usually pigmented, appendages robust, not elongated. Eyes present, pigmented. Head with dorsal yellow presumably photosensitive spots. Rostrum short, bluntly pointed. Lateral cephalic lobe with shallow inferior sinus. Antenna I longer than antenna II; accessory flagellum two-segmented; peduncular article I usually equal to article II. Antenna II with oval elongated calceoli in ♂♂; ♀♀ without calceoli. Incisor of right mandible four-dentate; lacinia mobilis and molar of both mandibles well developed; article II of palp longer than article III, bearing row of setae medially; palp article III without group of A-setae. Inner lobes of lower lip feebly developed. Coxal plates I–III deep; coxal plate IV deep, with excavation. Gnathopod I with trapezoid propodus, which is ~ 1.2'–1.4' longer than broad; palmar margin almost straight or slightly oblique, with double row of inner and outer bifurcate robust setae; palm groove (depression) feebly developed. Gnathopod II with subquadrate propodus, larger than in gnathopod I, stronger in ♂♂, ~ 1.5' longer than broad, palmar margin armed with double row of distally notched robust setae, palm groove (depression) feebly developed. Pereopod VI longer than pereopod VII; dactyli with 2–4 stiff strong setae (spines) on inner face. Urosomites completely fused (coalesced) without visible sutures. Coxal gills on pereopods II–VI or II–VII, gills VI and VII very small. Single, median and paired, simple sternal gills/processes present. Oostegites (brood plates) on pereopods II–IV large in ♀♀, ovoid, with long marginal setae, small oostegite present on pereopod V. Epimeral (pleonal) plates I–III with sharply produced posterior angles. Pleopods I–III subequal in length, peduncles with 2–5 coupling setae (retinacula) each. Uropod I with peduncular mostly vestigial distoventral distal serrate process in ♂♂. Uropod III squamiform, without rami, with several strong apical setae (spines). Telson subquadrate, ~ 1.2'–1.3' longer than wide, with apical notch, reaching ~ 1/3 of its length, lobes armed with seven or eight strong setae each. Sexual dimorphism could be marked or subtle; ♂♂ possess by sexually dimorphic antenna II, both gnathopods, uropod I and telson.

**Juveniles** (see Fig. 9). Generally similar to adults, with less developed armature of all appendages. Dactyli of pereopods elongated, simple, without strong setae on inner face. Pleopods with two coupling setae (retinacula) each. Uropod III squamiform, without rami, with single apical seta (spine). Telson oval, approx. as long as wide, with shallow distal notch, lobes with single strong spine each.

##### Included species.

*Caucasirella fontinalis* (Marin & Palatov, 2021), comb. nov. (type species of the genus), *Caucasirella shepsiensis* (Sidorov, 2015), comb. nov., *Caucasirella asheensis* (Palatov & Marin, 2021), c omb. nov., *Caucasirella fanagorica* (Palatov & Marin, 2021), comb. nov., *Caucasirella mikhailovi* (Marin & Palatov, 2021), comb. nov., *Caucasirella shapsuhica* sp. nov., *Caucasirella mzymtiensis* sp. nov. and *Caucasirella lazareviensis* sp. nov.

##### Etymology.

The genus is named after The Great Caucasian Mountain Ridge (Caucasus), where it is distributed in the northwestern foothills. Gender – feminine.

##### Differential diagnosis.

The new genus, *Caucasirella* gen. nov., is morphologically very similar to the Hyrcanian genus *Lyurella* Derzhavin, 1939 with the type species *Lyurella hyrcana* Derzhavin, 1939, described from coastal refugium of the southeastern Azerbaijan (after Derzhavin 1939; Sidorov 2015), while representatives of the genus are also present along the Iranian coasts of the Caspian Sea (Hyrcania) (Ruffo 1974). From *L. hyrcana*, it can be separated by 1) antenna I with peduncular article I usually equal to article II (vs longer than article II); 2) retinacules of pleopods with two or three coupling hooks (vs 4 hooks); 3) peduncular distoventral serrate process of uropod I in ♂♂ mostly vestigial (vs well-marked); and 4) distal notch of telson reaching to ~ 1/4–1/3 of its length (vs shallow distal notch).

By other features, the adult individuals of the genus could be separated from other Palaearctic crangonyctid genera by 1) the presence of well-developed pigmented eyes (present only in *Synurella* and *Eosynurella*); 2) mandibular palp article III without group of A-setae; 3) the presence of additional strong setae (spines) on dactyli of pereopods III–VII (present only in *Eosynurella*); and 4) completely fused urosomites without visible suture (fused only in *Synurella*). At the same time, *Eosynurella* could be separated by the presence of usually only two coupling hooks in retinacules and fused urosomites with a visible suture, while *Synurella* has simple dactyli of ambulatory pereopods, without additional strong setae (spines).

##### Distribution.

All species of *Caucasirella* gen. nov. are recently known from the various hypogean habitats (riverine hyporhea and springs) of the western foothills of the Great Caucasian Ridge (Caucasus) (see Sidorov 2015; Marin and Palatov 2021; present paper).

#### Caucasirella fanagorica

Taxon classificationAnimaliaAmphipodaCrangonyctidae

(Palatov & Marin, 2021) comb. nov.

0500F450-E02E-542C-AD15-7D804A0CF594

Fig. 2

##### New material examined.

Russian Federation, Krasnodar Territory, Goryachy Klyuch District: • 13♂♂, 29♀♀ (LEMMI) – Fanagoryiskaya Village, hyporhea of the Shpalnaya Shchel River, in a tributary of the Chepsi River, 44°30'48.2"N, 39°06'19.9"E, coll. D. Palatov & I. Marin, 26 March 2025; • 10♂♂, 8♀♀ (LEMMI) – Bezymannoe Village, hyporhea of the Orlova Shchel River, 44°33'04.4"N, 39°05'46.3"E, coll. D. Palatov & I. Marin, 26 March 2025; • 5♂♂, 8♀♀ (LEMMI) – Mirnyi Village, hyporhea of the Kol-Mitrofanova Shchel River, 44°36'12.3"N, 39°00'28.2"E, coll. D. Palatov & I. Marin, 26 March 2025; • 9♂♂, 11♀♀ (LEMMI) – Mirnyi Village, inside a small well, 44°36'21.0"N, 38°59'57.9"E, coll. D. Palatov & I. Marin, 26 March 2025; • 6♂♂, 8♀♀ (LEMMI) – Mirnyi Village, hyporhea of the Thamakhinskaya Shchel River, 44°32'56.4"N, 38°58'23.1"E, ~ 102 m a.s.l., coll. D. Palatov & I. Marin, 26 March 2025; • 5♂♂, 7♀♀ (LEMMI) – Goryacheklyuchevskoy State Nature Reserve, near Khrebtovoe Village, basin of the Kobza River, in a small forest stream flowing into the Tambovskaya Shchel River, 44°32'15.2"N, 38°58'00.2"E, ~ 171 m a.s.l., coll. D. Palatov & I. Marin, 29 March 2025; • 9♂♂, 10♀♀ (LEMMI) – basin of the Kobza River, Thamakhinskaya Shchel River, opposite Khrebtovoe village, 44°32'56.6"N, 38°58'18.07"E, ~ 160 m a.s.l., coll. D. Palatov & I. Marin, 30 March 2025.

##### Diagnosis.

Medium-sized species, body length up to 5.5 mm (♀♀) and 5.0 mm (♂♂), with expressed sexual dimorphism. Inner lobe of labium poorly developed. Antenna I with relatively long basal antennal article, which is ~ 2× longer than wide. Pereopod VI as long as pereopod VII; dactyli of pereopods III–VII with two or three stiff setae on inner face. Epimeral plates with 2–4 ventral setae. Coxal plate VII deep, with gill. Pleopod I with two coupling hooks in retinacula, with three coupling hooks in retinacules of pleopods II and III. Telson with deep apical notch, reaching ~ 1/3 of its length.

##### GenBank accession numbers.

MZ366352, MZ366353, PZ956239, PZ956240 (Chepsi River) and PZ956241, PZ956242 (Mirniy).

##### Coloration.

**Figure 2.**
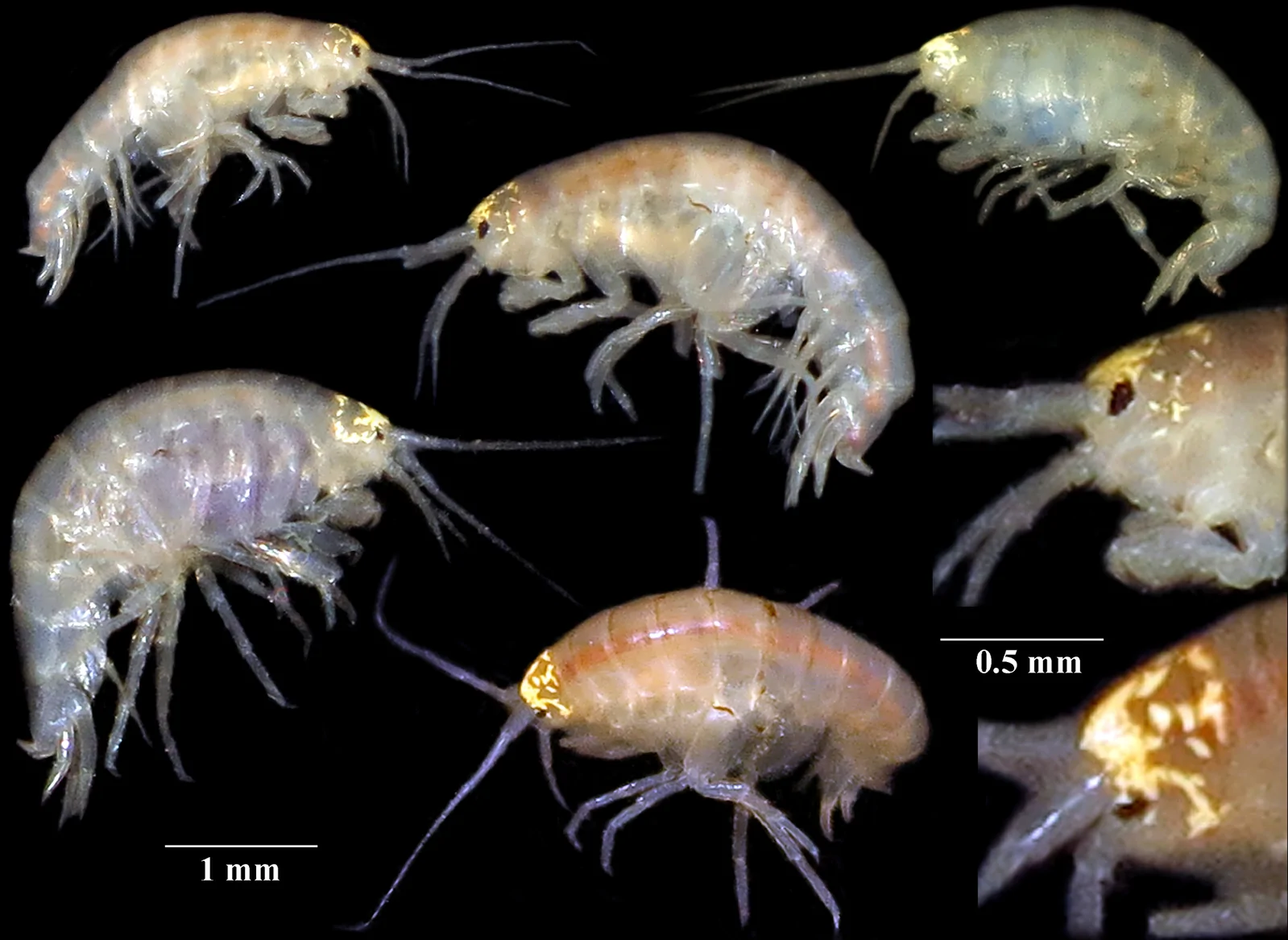
*Caucasirella fanagorica* (Palatov & Marin, 2021), comb. nov., general living coloration body and head.

The general coloration of the body and appendages is pale whitish, reddish or yellowish, the integument is transparent or translucent; eyes are black pigmented, well visible; yellow photosensitive spots occupy the front of the head, reaching the eyes, represented by separate or fused clusters (Fig. 2).

##### Ecology.

Riverine hyporheic species that lives in bottom habitats of the small rivers, springs, streams and their lower reaches, under rocks and large pebbles. Avoids sunlight, but does not burrow into the ground, is not a stygobiotic.

##### Distribution.

Currently, the species is known in various small streams and rivers located around the mountain Khrebtovaya (649 meters a.s.l., 44°32'51.7"N, 39°01'49.1"E), in the upper basin of the Psekups River (including right small tributaries, such as Kobza, Kavyrze, and Chepsi rivers), located in the Goryachy Klyuch District of the Krasnodar Krai (see Marin and Palatov 2021b; present study).

#### Caucasirella asheensis

Taxon classificationAnimaliaAmphipodaCrangonyctidae

(Palatov & Marin, 2021) comb. nov.

1CB58255-59B7-50E4-A1E5-A5ECC520929A

Figs 3, 4

##### New material examined.

• 1♂, 12♀♀ (LEMMI) – Russian Federation, Krasnodar Territory, Sochi city district, Lazarevskoe, in a small puddle-well on the outskirts of the Stroitel village, located on the right bank of the Ashe River, 43°59'41.2"N, 39°18'31.6"E, ~ 66 m a.s.l., coll. D. Palatov & I. Marin, 6 June 2025.

##### Diagnosis

**Figure 3.**
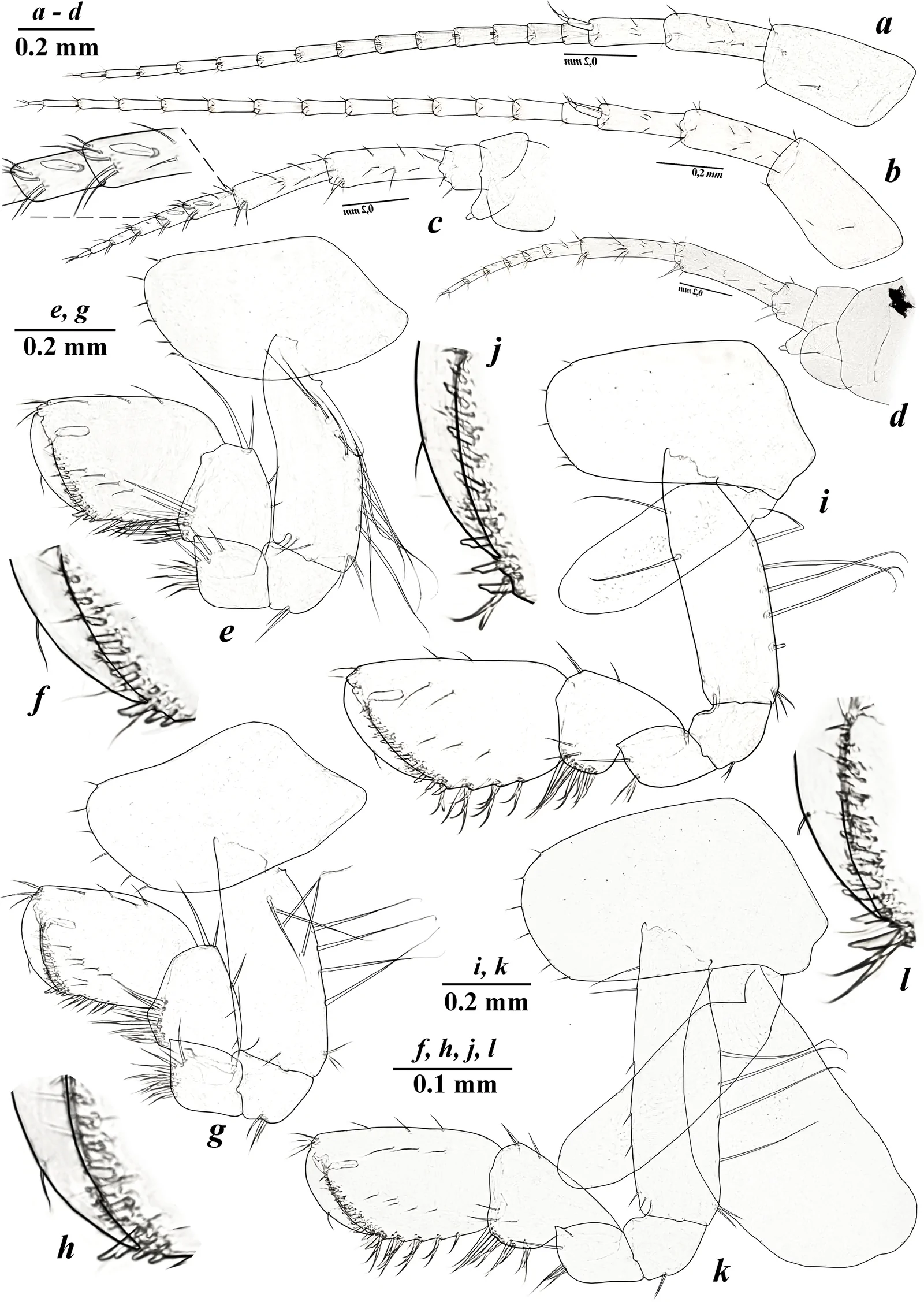
*Caucasirella asheensis* (Palatov & Marin, 2021), comb. nov. from Stroitel village, Lazarevskoe, ♂ (LEMMI) (**a, c, e, f, i, j**) and ♀ (LEMMI) (**b, d, g, h, k, l**): **a, b**. Antenna I; **c, d**. Antenna II; **e, g**. Gnathopod I; **f, h**. Palmar margin of propodus (palm) of gnathopod I; **i, k**. Gnathopod II; **j, l**. Palmar margin of propodus (palm) of gnathopod II.

**Figure 4.**
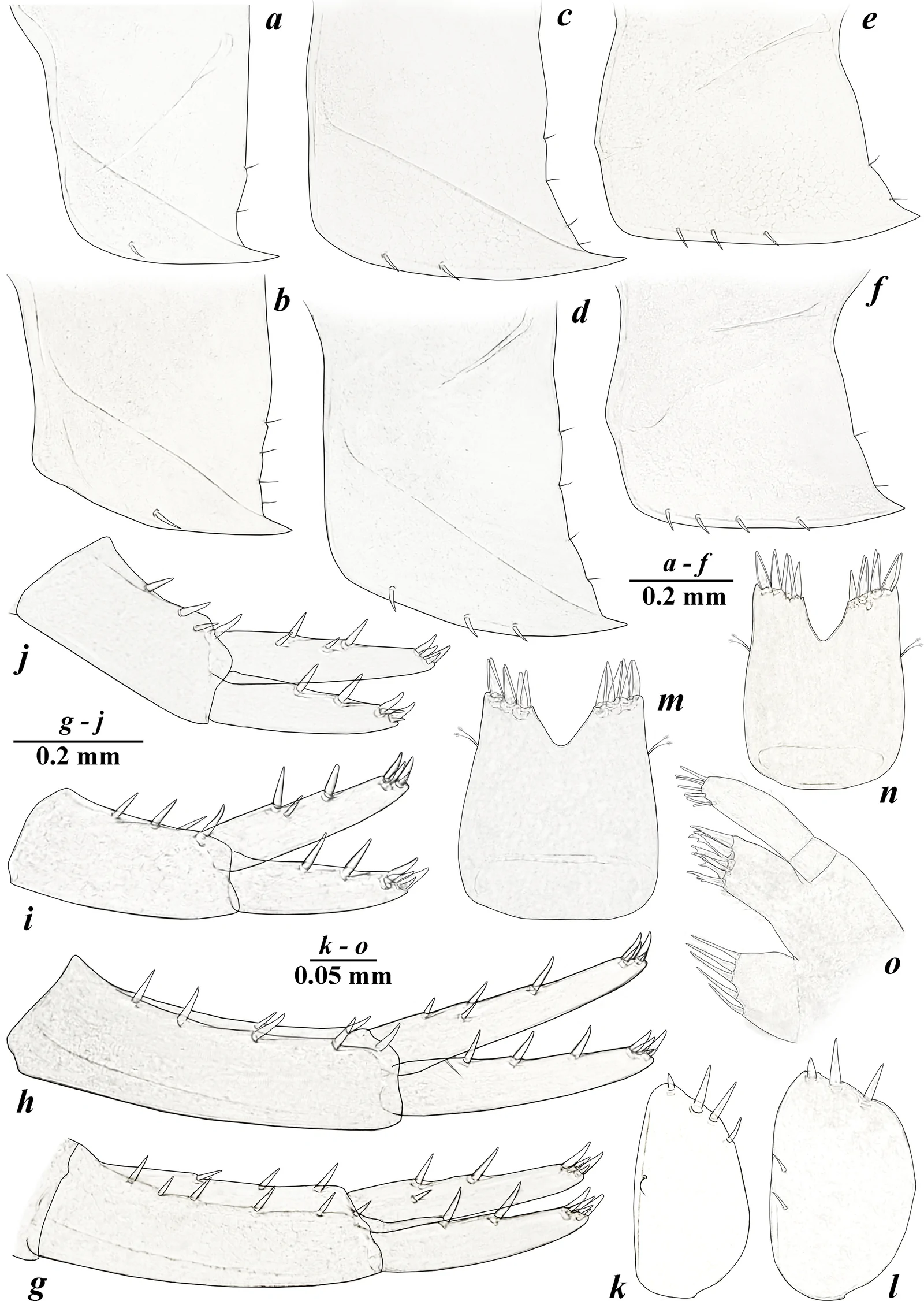
*Caucasirella asheensis* (Palatov & Marin, 2021), comb. nov. from Stroitel village, Lazarevskoe, ♂ (LEMMI) (**a, c, e, g, i, k, n, o**) and ♀ (LEMMI) (**b, d, f, h, j, l, m**): **a, b**. Epimeral plate I; **c, d**. Epimeral plate II; **e, f**. Epimeral plate III; **g, h**. Uropod I; **i, j**. Uropod II; **k, l**. Uropod III; **m, n**. Telson; **o**. Maxilla I.

(Figs 3, 4). Medium-sized species, body length up to 6.2 mm (♀♀) and 5.6 mm (♂♂), with expressed sexual dimorphism. Inner lobe of labium poorly developed. Antenna I with relatively long basal antennal article, which is ~ 2' longer than wide. Calceoli on antenna II well-marked in ♂♂. Pereopod VI as long as pereopod VII; dactyli of pereopods with 2–4 stiff setae on inner face. Epimeral plates with 2–4 ventral setae. Coxal plate V with feeble developed anterior lobe of in ♀♀. Coxal plate VII deep, with gill. Pleopod I with two coupling hooks in retinacula, with three coupling hooks in retinacules of pleopods II–III. Telson with deep apical notch, reaching ~ 1/3 of its length.

##### GenBank accession numbers.

MZ366360–MZ366363 and PZ956243, PZ956244 (Stroitel).

##### Distribution and ecology.

Currently, the species is known only from two localities ~ 5 km apart, in small underground streams on the banks of the Ashe River (44°0'55.87"N, 39°21'54.09"E and 43°59'41.2"N, 39°18'31.6"E) (see Marin and Palatov 2021b). Hypogean/stygobiotic species that lives in small springs, streams and their lower reaches, under rocks and large pebbles. Avoids sunlight, but does not burrow into the ground, is not a stygobiotic. Not found in the stream and hyporheic habitats of the Ashe River.

#### Caucasirella fontinalis

Taxon classificationAnimaliaAmphipodaCrangonyctidae

(Marin & Palatov, 2021) comb. nov.

90C318B5-F97F-5205-A5F9-3002763115E0

##### New material examined.

Russian Federation, Krasnodar Territory, Sochi city district, Lazarevskoe, Maryinskoe roadway: • 3♂♂, 24♀♀ (LEMMI) – in a small stream on the left bank of the Psezuapse River, opposite the village of Tatyanovka, 43°57'18.9"N, 39°25'13.0"E, coll. D. Palatov & I. Marin, 03 June 2025; • 2♂♂, 8♀♀ (LEMMI) – in a small stream on the left bank of the Psezuapse River, opposite the village of Tatyanovka, 43°57'18.1"N, 39°25'15.2"E, coll. D. Palatov & I. Marin, 03 June 2025.

##### Diagnosis.

Medium-sized species, body length up to 6.0 mm (♀♀) and 4.5 mm (♂♂), with expressed sexual dimorphism. Calceoli on antenna II small and less marked (mostly transparent). Inner lobe of labium poorly developed. Pereopod VI as long as pereopod VII; dactyli of pereopods III–VII with four or five stiff setae on inner face. Epimeral plates I–II with one or two ventral setae. Coxal plate V with feeble developed anterior lobe in ♀♀. Coxal plate VII deep, with gill. Pleopods I–III with three coupling hooks in retinacules. Telson with apical notch reaching ~ 1/3–1/4 of its length, each lobe armed with seven robust spines and two additional submarginal plumose setae.

##### GenBank accession numbers.

MZ366354–MZ366357 (Makopse) and PZ956245, PZ956246 (Tatyanovka, Psezuapse).

##### Distribution and ecology.

Currently, the species is known only from two localities ~ 16 km apart, in a stream on the banks of the Makopse River (44°01'46.5"N, 39°15'08.5"E) (see Marin and Palatov 2021b) and two locations on the left bank of the Psezuapse River (43°57'18.9"N, 39°25'13.0"E and 43°57'18.1"N, 39°25'15.2"E) (present paper). Riverine Probably, hypogean species that lives in small springs, streams and their lower reaches, under rocks and large pebbles. Avoids sunlight, but does not burrow into the ground, is not a stygobiotic. Not found in the streams and riverine hyporheic habitats of the Makopse and Psezuapse rivers.

#### Caucasirella shapsuhica sp. nov.

Taxon classificationAnimaliaAmphipodaCrangonyctidae

63C31DFD-660E-5BB4-817C-FB6D4800616F

https://zoobank.org/1221156A-75B2-4A69-8C7D-10E6D1DAA955

Figs 5, 6, 7, 8

##### Material examined.

***Holotype***. • ♂ (bl. 5.2 mm) (ZMMU Mb-1321) – Russian Federation, Krasnodar Krai, Tuapse Urban Okrug, in the spring seepage in unnamed stream valley (a tributary of Shapsuho River), south of Moldavanovka village, 44°27'49.86"N, 38°53'23.87"E, coll. D. Palatov & I. Marin, 31 March 2025. ***Paratype***. • ♀ (bl. 6.5 mm) (ZMMU Mb-1322) – same locality and data as holotype.

##### Other material.

Russian Federation, Krasnodar Krai, Tuapse Urban Okrug: • 1♂, 4♀♀ (LEMMI) – in the riverine hyporhea of the small tributary of Shapsuho River, Moldavanovka village, 44°28'24.9"N, 38°53'02.2"E, coll. D. Palatov & I. Marin, 26 March 2025; Krasnodar Krai, Goryachy Klyuch Urban Okrug: • 3♂♂, 12♀♀ (LEMMI) – in the stream from a well in Mirny settlement, Thamashinka River valley, 44°36'20.96"N, 38°59'57.94"E, ~ 102 m a.s.l., coll. D. Palatov & I. Marin, 26 March 2025; Krasnodar Krai, Goryachy Klyuch Urban Okrug: • 23♂♂, 49♀♀ (LEMMI) – in the stream (left tributary of the Chepsi River) within the boundaries of Fanagoriyskoye village, 44°30'48.12"N, 39°06'21.34"E, ~ 98 m a.s.l., coll. D. Palatov & I. Marin, 27 March 2025; Krasnodar Krai, Goryachy Klyuch Urban Okrug: • 10♂♂, 75♀♀ (LEMMI) – in the temporary stream (left tributary of the Tambovskaya Shchel River), 44°32'16.09"N, 38°57'58.79"E, ~ 171 m a.s.l., coll. D. Palatov & I. Marin, 29 March 2025; Krasnodar Krai, Goryachy Klyuch Urban Okrug: • 19♂♂, 31♀♀ (LEMMI) – in the Thamakhinskaya Shchel River opposite Khrebtovoe village, 44°32'56.6"N, 38°58'18.07"E, ~ 160 m a.s.l., coll. D. Palatov & I. Marin, 30 March 2025; Krasnodar Krai, Tuapse Urban Okrug: • 1♂, 6♀♀ (LEMMI) – in the stream north of the centre of Moldavanovka settlement, on the opposite side of the highway, 44°28'19.07"N, 38°52'58.27"E, ~ 160 m a.s.l., coll. D. Palatov & I. Marin, 30 March 2025; Krasnodar Krai, Tuapse Urban Okrug: • 12♀♀ (LEMMI) – in the Gornar River south of Moldavanovka settlement, 44°27'57.5"N, 38°53'14"E, ~ 160 m a.s.l., coll. D. Palatov & I. Marin, 31 March 2025; Krasnodar Krai, Tuapse Urban Okrug: • 7♂♂, 30♀♀ (LEMMI) – in the Shchel Nizhny Sad (a tributary of Shapsuho River), right stream tributary of the axial river, 44°28'32.76"N, 38°48'39.41"E, ~ 113 m a.s.l., coll. D. Palatov & I. Marin, 1 April 2025.

##### Description.

***Body***: moderately stout, stygomorphic.

**Figure 5.**
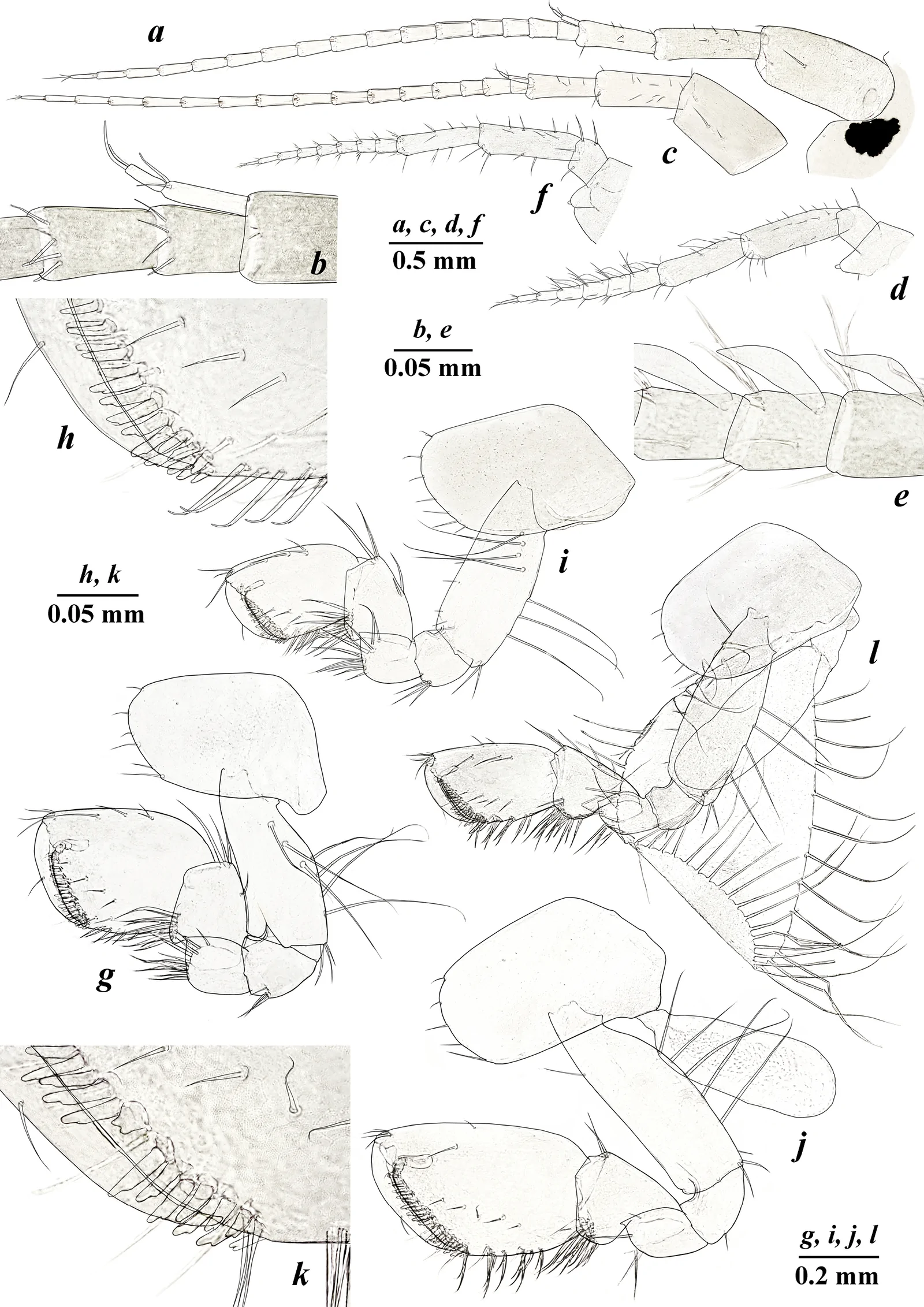
*Caucasirella shapsuhica* sp. nov. from the upper reach of the Shapsuho River, holotype ♂ (ZMMU Mb-1321) (**a, b, d, e, g, h, j, k**), paratype ♀ (ZMMU Mb-1322) (**c, f, i, l**): **a, c**. Antenna I; **b**. Accessory flagellum of antenna I; **d, f**. Antenna II; **e**. Calceoli on flagellum of antenna II; **g, i**. Gnathopod I; **h**. Pa lmar margin of propodus (palm) of gnathopod I; **j, l**. Gnathopod II; **k**. Palmar margin of propodus (palm) of gnathopod II.

***Head***: large, smooth, without rostrum, with subrounded lateral cephalic lobes and excavated anteroventral sinus; eyes (ommatidia) well seen on anterior lobe (Fig. 5a); inter-antennal lobe wide, bluntly rounded anteriorly.

***Antenna I*** (Fig. 5a, c) ~ 46–48% of body length, ~ 1.8' longer than antenna II; primary flagellum with 16 segments in ♂♂ and ♀♀, with aesthetascs on distal segments; accessory flagellum two-segmented, distal segment ~ 2× shorter than basal one.

***Antenna II*** (Fig. 5d, f): gland cone distinct, distally pointed; peduncle ~ 1.5' longer than flagellum, with robust setae tightly covering segments III and IV, peduncle of segment IV ~ 1.2' longer than segment V; flagellum seven (♂♂) or eight (♀♀)-segmented; with calceoli on peduncle and flagellum in ♂♂ (Fig. 5e).

**Figure 6.**
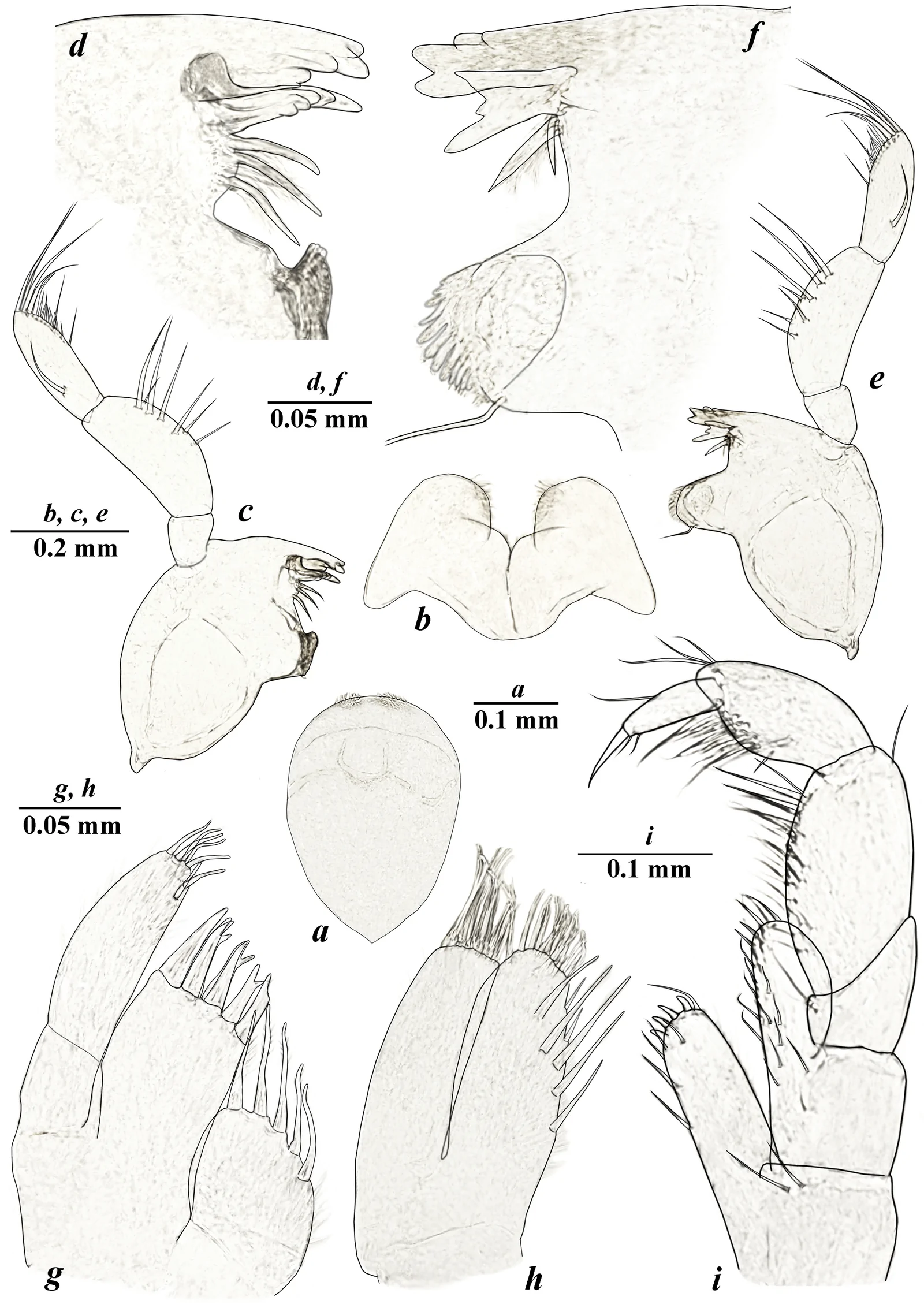
*Caucasirella shapsuhica* sp. nov. from the upper reach of the Shapsuho River, holotype ♂ (ZMMU Mb-1321): **a**. Upper lip; **b**. Lower lip; **c**. Left mandible; **d**. Same, incisor process and pars incisiva; **e**. Right mandible; **f**. Same, incisor process and pars incisiva; **g**. Maxilla I; **h**. Maxilla II; **i**. Maxilliped.

***Mandible*** (Fig. 6c–f): left mandible (Fig. 6c, d) incisor five-dentate, lacinia mobilis five-dentate, with three robust plumose accessory setae, molar process with one seta; right mandible (Fig. 6e, f) incisor four-dentate, lacinia mobilis trifurcate, both lobes with numerous protuberances; underlying with row of two robust plumose setae; molar process similar to left mandible; palp three-segmented, segment II with seven setae; segment III ~ 2.9' longer than wide, with straight margins, with seven or eight separate D-setae, one C-seta, one B-seta and four separate E-setae.

***Labrum*** (upper lip) (Fig. 6a): oval, apical margin with numerous small fine setae.

***Labium*** (lower lip) (Fig. 6b): inner lobes feebly developed.

***Maxilla I*** (Fig. 6g): inner plate with five plumose marginal setae, outer plate with seven apical comb-spines; palp two-segmented, distal segment pubescent, ~ 2.2' of basal segment, apical margin of distal segment with seven simple setae.

***Maxilla II*** (Fig. 6h): inner, outer plates covered in pubescent setae; outer plate smaller than inner plate, weakly narrowing distally, with 13 apical setae; inner plate narrowing slightly distally, with group of dense short setae on apex, with oblique row of five short plumose setae.

***Maxilliped*** (Fig. 6i): inner plate much shorter than outer plate, with four spines and five simple setae apically and one spine subapically, outer plate narrow, with double row of 20 medial stiff setae of different lengths, palp quadriarticulate, article I with two setae on outer margin, article II with row of 20 simple setae on inner margin and one seta on outer margin, article III sub-quadrate; dactylus with one seta on outer margins, nail long with two thin setae at hinge.

***Gnathopod I*** (Fig. 5g, i): smaller than gnathopod II in ♂♂ and ♀♀: coxal plate sub-rectangular, distally rounded, with six apical and numerous facial setae, width/depth ratio ~ 0.70'; basis width/length ratio close to 0.4/1, without setae on anterior margin, three long setae on inner face and four long setae on posterior margin; ischium approx. as long as wide, with tuft of posterodistal setae; merus quadrate, approx. as long as wide, with 12 distal setae; carpus ~ 0.56' of length of basis and ~ 0.62' of length of propodus, with three large serrated setae in inner margin and seven serrated setae in outer margin; propodus ~ 1.3' longer than broad in ♂♂ (Fig. 5g) and ~ 1.5' longer than broad in ♀♀ (Fig. 5i), with four simple setae in anterior margin and six serrated setae in posterior margin; distal margin of palm slightly oblique in ♂♂ and ♀♀, with double row of 6 inner and seven outer bifurcate robust setae (Fig. 5h); palm groove (depression) feebly developed, with three inner and four outer robust setae (Fig. 5h); dactylus with one outer seta.

***Gnathopod II*** (Fig. 5j, l): coxal plate sub-rectangular, with 5–7 apical and numerous facial setae, width/depth ratios ~ 0.6'; basis width/length ratio close to 0.34/1, with several (4 or 5) long setae inserted along posterior margin and one or two short simple setae in anterior margin; ischium approx. as long as wide, with tuft of posterodistal setae; merus quadrate, approx. as long as wide, with four or five distal setae; carpus ~ 0.50' of length of basis and ~ 0.56' of propodus, with distal anterior and distal posterior groups of plumose setae; propodus ~ 1.56' longer than broad in ♂♂ (Fig. 5j) and ~ 1.62' longer than broad in ♀♀ (Fig. 5l), with one or two simple anterior setae, 1–3 superior medial, five inferior medial and four or five groups of posterior setae; palm slightly oblique in ♂♂ and ♀♀, with double–row of seven inner and seven outer bifurcate robust setae; palm groove (depression) feebly developed, with four inner and three outer robust setae (Fig. 5k); dactylus with one outer seta.

**Figure 7.**
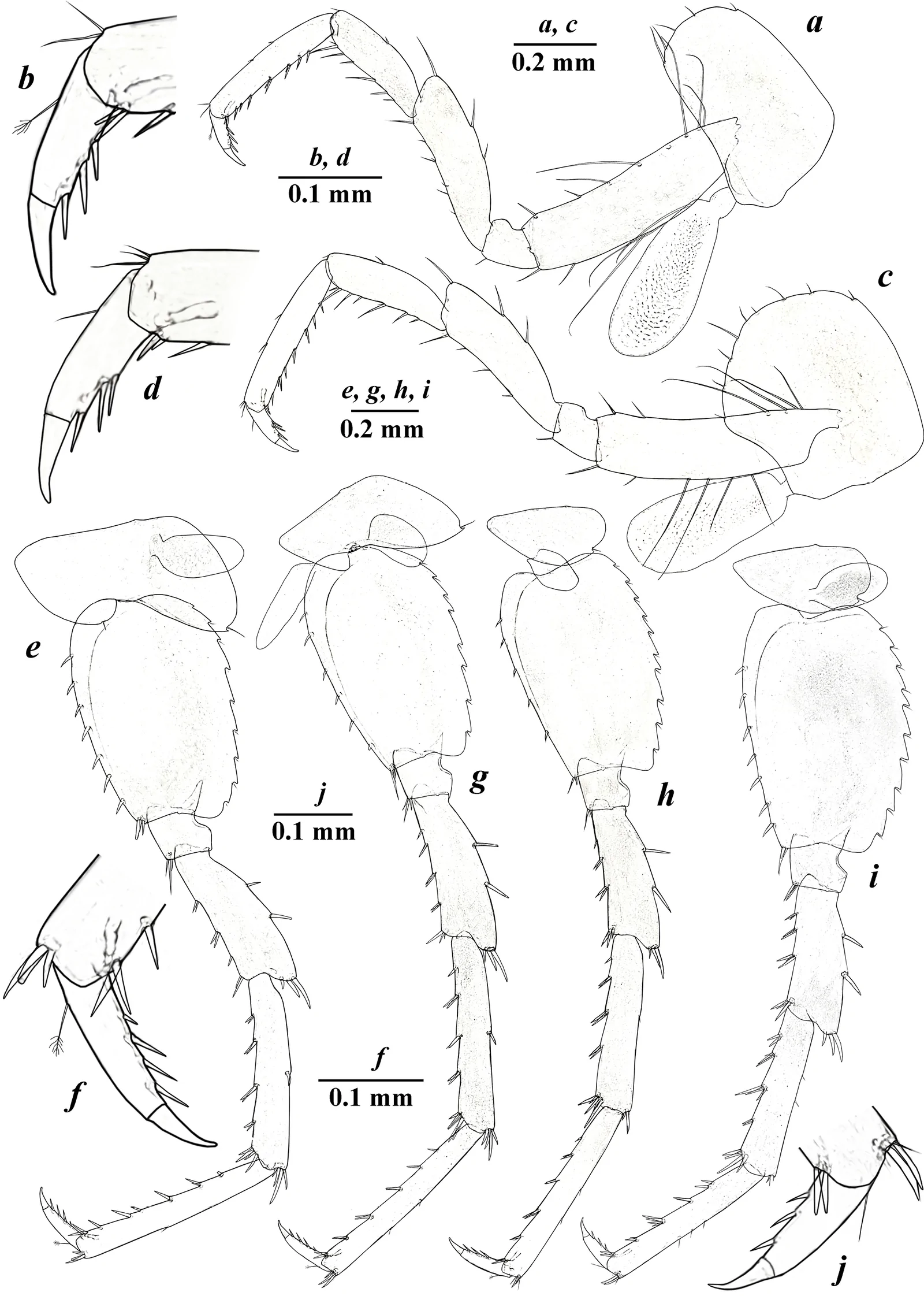
*Caucasirella shapsuhica* sp. nov. from the upper reach of the Shapsuho River, holotype ♂ (ZMMU Mb-1321) (**a–h**), paratype ♀ (ZMMU Mb-1322) (**i, j**): **a**. Pereopod III; **b**. Dactylus of pereopod III; **c**. Pereopod IV; **d**. Dactylus of pereopod IV; **e**. Pereopod V; **f**. Dactylus of pereopod V; **g**. Pereopod VI; **h, i**. Pereopod VII; **j**. Dactylus of pereopod VII.

***Pereopod III*** (Fig. 7a): similar in ♂♂ and ♀♀, coxal plate sub-rectangular, with five apical and numerous facial setae, width/depth ratio ~ 0.5'; basis ~ 3.7' as long as wide, with long anterior and posterior simple setae; ischium approx. as long as wide, with one distal seta; merus ~ 3.5' longer than wide, ~ 0.70' of basis, ~ 1.40' of carpus and mostly equal to propodus in length; carpus ~ 3.3' longer than wide, ~ 0.82' of propodus in length; propodus ~ 5.5' longer than wide, with six or seven ventral spine-like setae; dactylus (Fig. 7b) ~ 0.4' of propodus, relatively stout, with plumose seta on outer margin and three strong stiff setae on inner face.

***Pereopod IV*** (Fig. 7c): similar in ♂♂ and ♀♀, coxal plate oval approx. as long as wide, with eight or nine apical and numerous facial setae; basis ~ 4.0' as long as wide, with long anterior and posterior simple setae; ischium approx. as long as wide, with one distal seta; merus ~ 3.7' longer than wide, ~ 0.7' of basis, ~ 1.3' of carpus and approx. mostly equal to propodus in length; carpus ~ 3.5' longer than wide, ~ 0.80' of propodus in length; propodus ~ 5.7' longer than wide, with seven or eight ventral spine-like setae; dactylus (Fig. 7d) ~ 0.4' of propodus, relatively stout, with plumose seta on outer margin and three strong stiff setae on inner face.

***Pereopods V*–*VII***: length ratio of pereopods V/VI/VII close to 1/1.15/1.02 (♂♂) and 1/1.12/1.06 (♀♀).

***Pereopod V*** (Fig. 7e): mostly similar in ♂♂ and ♀♀, coxal plate large, bilobate with distinct anterior and posterior lobes; anterior lobe with one margin simple seta, posterior lobe with one margin stout seta, each with numerous facial setae; basis ~ 1.5' as long as wide, with numerous facial setae, posterior margin slightly convex, armed with nine shallow serrations, with bluntly convex distal corner, anterior margin with seven split-tipped robust and two distal setae; ischium approx. as long as wide, with three distal anterior setae; merus ~ 2.9' longer than wide, expanding posteriorly, 0.75' of basis, ~ 0.87' of carpus and ~ 0.80' of propodus in length; carpus ~ 5.0' longer than wide, ~ 0.9' of propodus in length; propodus ~ 8.1' longer than wide, with five or six ventral spine-like setae; dactylus (Fig. 7f) ~ 0.3' of propodus, with plumose seta on outer margin and four strong stiff setae on inner face.

***Pereopod VI*** (Fig. 7g): mostly similar in ♂♂ and ♀♀, coxal plate bilobate, with distinct posterior and vestigial anterior lobes; anterior lobe without setae, posterior lobe with one margin stout seta, each with numerous facial setae; basis ~ 1.6' as long as wide, with numerous facial setae, posterior margin slightly convex, armed with eight shallow serrations, with bluntly convex distal corner, anterior margin with six split-tipped robust and two distal setae; ischium approx. as long as wide, with four distal anterior setae; merus ~ 3.2' longer than wide, expanding posteriorly, ~ 0.74' of basis and carpus and ~ 0.84' of propodus in length; carpus ~ 5.8' longer than wide, ~ 0.98' of propodus in length; propodus ~ 7.8' longer than wide, with five or six ventral spine-like setae; dactylus ~ 0.3' of propodus, with plumose seta on outer margin and four strong stiff setae on inner face.

***Pereopod VII***. Male (Fig. 7h): coxal plate small, semi-lunar, with one posterior seta; basis ~ 1.47' as long as wide, with numerous facial setae, posterior margin convex, triangularly produced, armed with ten shallow serrations, with bluntly convex distal corner, anterior margin with five split-tipped robust and two distal setae; ischium approx. as long as wide, with three distal anterior setae; merus ~ 3.0' longer than wide, expanding posteriorly, ~ 0.72' of basis, ~ 0.86' of carpus and ~ 0.79' of propodus in length; carpus ~ 4.9' longer than wide, ~ 0.9' of propodus in length; propodus ~ 7.3× longer than wide, with five ventral spine-like setae; dactylus ~ 0.3' of propodus, with plumose seta on outer margin and three strong stiff setae on inner face. Female (Fig. 7i): coxal plate small, semi-lunar, with one posterior seta; basis ~ 1.5' as long as wide, with numerous facial setae, posterior margin significantly convex, armed with ten shallow serrations, with bluntly convex distal corner, anterior margin with seven split-tipped robust and 3 distal setae; ischium approx. as long as wide, with three distal anterior setae; merus ~ 2.7' longer than wide, expanding posteriorly, ~ 0.65' of basis and carpus and 0.86' of propodus in length; carpus ~ 4.3' longer than wide, ~ 0.96' of propodus in length; propodus ~ 6.9' longer than wide, with four or five ventral spine-like setae; dactylus ~ 0.3' of propodus, with plumose seta on outer margin and three strong stiff setae on inner face (Fig. 7j).

***Gills, brood plates***: coxal gills on somites II–VII, somites V–VII with lanceolate sternal gills. Male: coxal gills II–VII ovoid, gills/bases pereopod ratios ~ 0.82/1, 0.73/1, 0.68/1, 0.37/1, 0.29/1 and 0.24/1, respectively. Female: coxal gills II–VII ovoid, gills/bases pereopod ratios of ~ 0.96/1, 0.99/1, 1.01/1, 0.49/1, 0.43/1 and 0.32/1, respectively. Slender, setaceous brood plates on somites II–V, decreasing in size posteriorly.

**Figure 8.**
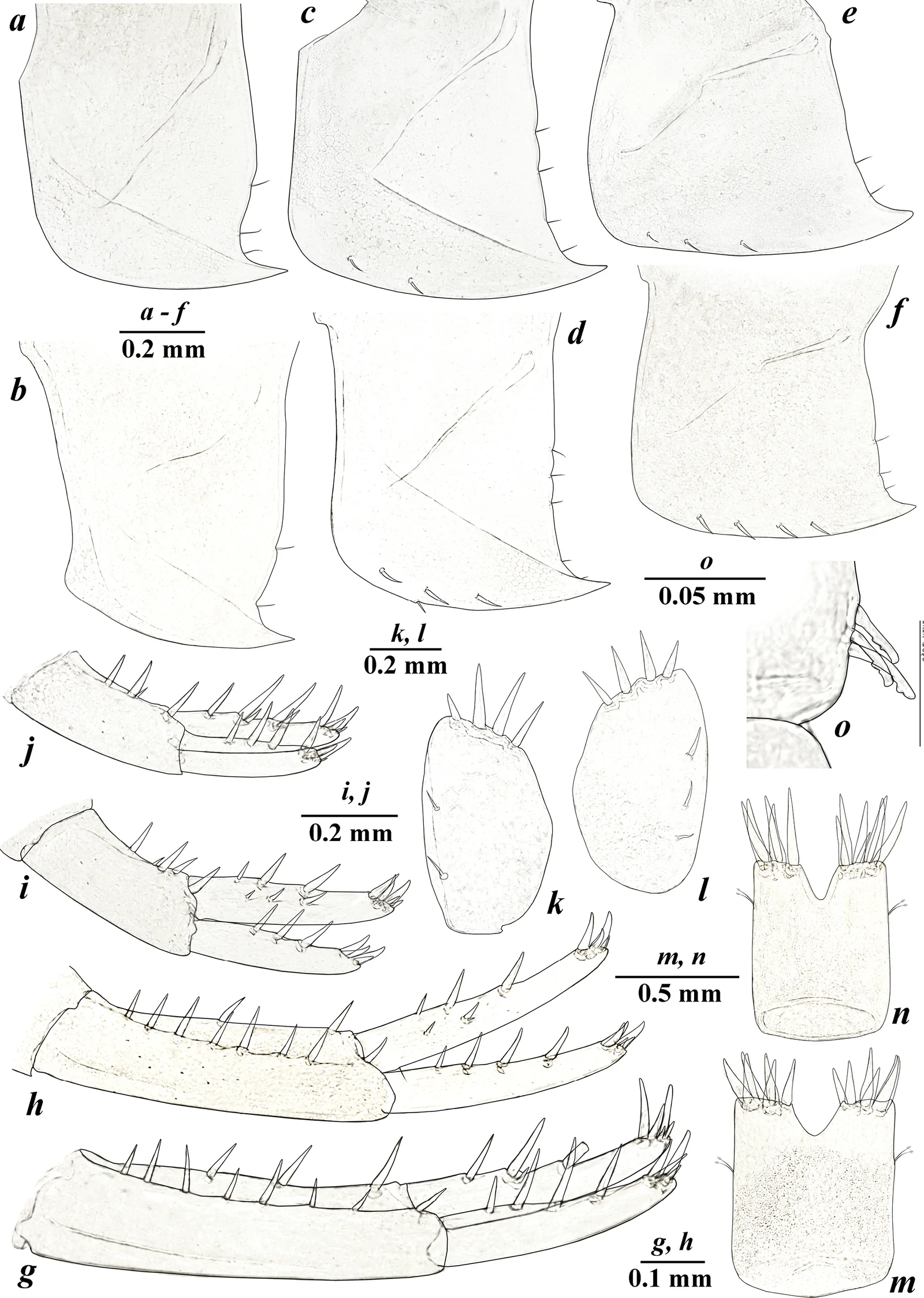
*Caucasirella shapsuhica* sp. nov. from the upper reach of the Shapsuho River, holotype ♂ (ZMMU Mb-1321) (**a, c, e, g, i, k, m, o**), paratype ♀ (ZMMU Mb-1322) (**b, d, f, h, j, l, n**): **a, b**. Epimeral plate I; **c, d**. Epimeral plate II; **e, f**. Epimeral plate III; **g, h**. Uropod I; **i, j**. Uropod II; **k, l**. Uropod III; **m, n**. Telson; **o**. Coupling hooks of retinacula of pleopod III.

Pleopods: peduncle with three coupling hooks in retinacules (Fig. 8o) of both ♂♂ and ♀♀; outer and inner rami with seven or eight and eight segments, respectively; basal segment of outer ramus without clothes-pin setae.

***Epimera***: Epimeral plate I (Fig. 8a) distally produced and strongly sharpened, ventral margin without spines, posterior margin with two or three setae. Epimeral plate II (Fig. 8b) distally produced and strongly sharpened, ventral margin armed with two or three spines, posterior margin with four or five setae. Epimeral plate III (Fig. 8c) distally produced and strongly sharp, ventral margin armed with three or four spines, posterior margin with two or three setae.

***Urosomites*** completely fused, smooth.

***Uropod I*** (Fig. 8g, h). Male: peduncle ~ 4.3' as long as wide (Fig. 8g); without distal marginally serrate process, with dorso-internal row of two robust spines, one subdistal spine and eight dorso-external robust spines; exopodite slightly shorter than endopodite; endopodite not paddle-like, with four dorsolateral and four apical spines; exopodite with three dorsolateral and four apical spines. Female (Fig. 8h): peduncle ~ 4.0' as long as wide, without distal serrate process; with dorso-internal row of one or two robust spines, one subdistal spine and eight dorso-external robust spines; exopodite mostly equal to endopodite; endopodite not paddle-like, with three dorsolateral and five apical and two ventral spine-like setae; exopodite with four dorsolateral and four apical spines.

***Uropod II*** (Fig. 8i, j): peduncle ~ 2.4' as long as wide, subequal in length to endopodite and exopodite, with two or three outer robust, one inner robust and one or two subdistal spines; exopodite slightly shorter than endopodite, with five outer and four apical robust spines; endopodite with three outer and four apical robust spines.

***Uropod III*** (Fig. 8k, l): vestigial, squamiform, slightly longer than telson, with four short spiniform setae apically and two setules on dorsal face.

***Telson*** (Fig. 8m, n): close to rectangular, ~ 1.32' (♂♂) – 1.22' (♀♀) as long as broad; distal margin with relatively deep V-shaped notch, ~ 0.18'–0.21' of total length, each lobe armed with 5–7 robust spiniform setae, with two additional submarginal plumose setae.

##### Coloration.

The body and appendages yellowish or grayish transparent; well-pigmented black eyes well seen.

##### Size.

The largest collected ♂ has bl. 5.2 mm, ♀ – 6.5 mm.

##### GenBank accession numbers.

PZ956251, PZ956252, PZ956253 (Moldavanovka), PZ956254, PZ956255 (Gornar River) and PZ956256, PZ956257 (Schel Niznyi Sad).

##### Etymology.

The species is named after the Shapsuho River, where it was discovered.

##### Taxonomic remarks.

From the western Caucasian representatives of the genus *Caucasirella* gen. nov., mostly separated by shape and armature of both gnathopods and ambulatory pereopods, number of coupling hooks in retinacules of pleopods, armature of epimeral plate and shape of the telson.

From *C. shepsiensis* comb. nov. (after Sidorov 2015), the new species can be separated by 1) stouter propodus (palm) of gnathopod II, especially in ♂♂; 2) much anterior and posterior spine-like setae on basis of ambulatory pereopods (10 vs 8 spine-like setae); 3) three ventral spines on epimeral plate II and four ventral spines on epimeral plate III (vs 5 spines and 5 or 6 spines, respectively); 4) longer uropod I, and especially longer their peduncle; and 5) relatively deeper notch on telson.

From *C. mikhailovi* comb. nov. (see Marin and Palatov 2022), the new species can be separated by 1) stouter propodus (palm) of gnathopod I; 2) the presence of three or four additional spines on dactyli of ambulatory pereopods (vs 2 spines); 3) much anterior and posterior spine-like setae on basis of ambulatory pereopods (7 and 10, respectively vs 6 and 4 spine-like setae); 4) three coupling hooks in retinacules of pleopods (vs 2 hooks); 5) pointing back posteroventral angle of epimeral plate I (vs pointing back and down); 6) three ventral spines on epimeral plate II and four ventral spines on epimeral plate III (vs 4 and 3 spines, respectively); and 7) relatively deeper notch on telson.

From *C. fanagorica* comb. nov. (see Marin and Palatov 2022), the new species can be separated by 1) less oblique palmar margin of gnathopod II; 2) the presence of three or four additional spines on dactyli of ambulatory pereopods (vs 2 spines on PIII–PV); 3) much posterior spine-like setae on basis of ambulatory pereopods (10 vs 8 spine-like setae); and 4) relatively deeper notch on telson.

From *C. fontinalis* comb. nov. (see Marin and Palatov 2022), the new species can be separated by 1) longer propodus (palm) of gnathopod I, especially in ♀♀; 2) the presence of three or sometimes four additional spines on dactyli of ambulatory pereopods (vs always 4 or 5 spines); 3) much posterior spine-like setae on basis of ambulatory pereopods (10 vs 6 or 7 spine-like setae); 4) three ventral spines on epimeral plate II and four ventral spines on epimeral plate III (vs 2 spines and 2–4 spines, respectively); and 5) relatively deeper notch on telson.

From *C. asheensis* comb. nov. (see Marin and Palatov 2022; present paper), the new species can be separated by 1) stouter propodus (palm) of gnathopod II, especially in ♂♂; 2) much anterior and posterior spine-like setae on basis of ambulatory pereopods (10 vs 6 or 7 spine-like setae); 3) three ventral spines on epimeral plate II and 4 ventral spines on epimeral plate III (vs 2 spines and 3 spines, respectively); 4) longer uropod I, and especially longer their peduncle; and 5) relatively shallower notch on telson (vs notch deep, ~ 1/4–1/3 of telson length).

For the differences from *C. lazareviensis* sp. nov. and *C. mzymtiensis* sp. nov. see below.

##### Distribution and ecology.

The species is known only from the riverine hyporheic habitats of the upper reach of the small mountainous Shapsuho River, which flows from the southwestern slope of the Great Caucasus Mountains into the Black Sea near the Lermontovo village (44°18'08.5"N, 38°44'38.3"E).

#### Caucasirella lazareviensis sp. nov.

Taxon classificationAnimaliaAmphipodaCrangonyctidae

61BB78E3-3AAE-5693-86D9-C958A3BF4348

https://zoobank.org/E9C108AA-3217-4F25-B01B-7BBA993FE07F

Figs 9, 10, 11

##### Material examined.

***Holotype***, • ♂ (bl. 5.0 mm) (ZMMU Mb-1323) – Russian Federation, Krasnodar Krai, Sochi Urban Area, Lazarevsky district, in a small left tributary of the Psezuapse River flowing near the Maryino village, near the bus stop and the café, 43°56'30.95"N, 39°28'38.28"E, ~ 196 m a.s.l., coll. D. Palatov & I. Marin, 03 June 2025. ***Paratype***. • ♀ (bl. 5.5 mm) (ZMMU Mb-1324) – same locality and data as holotype.

##### Other material.

18♂♂, 38♀♀ (LEMMI) – same locality and data as holotype.

##### Description.

***Body***: moderately stout, stygomorphic (Fig. 13a).

**Figure 9.**
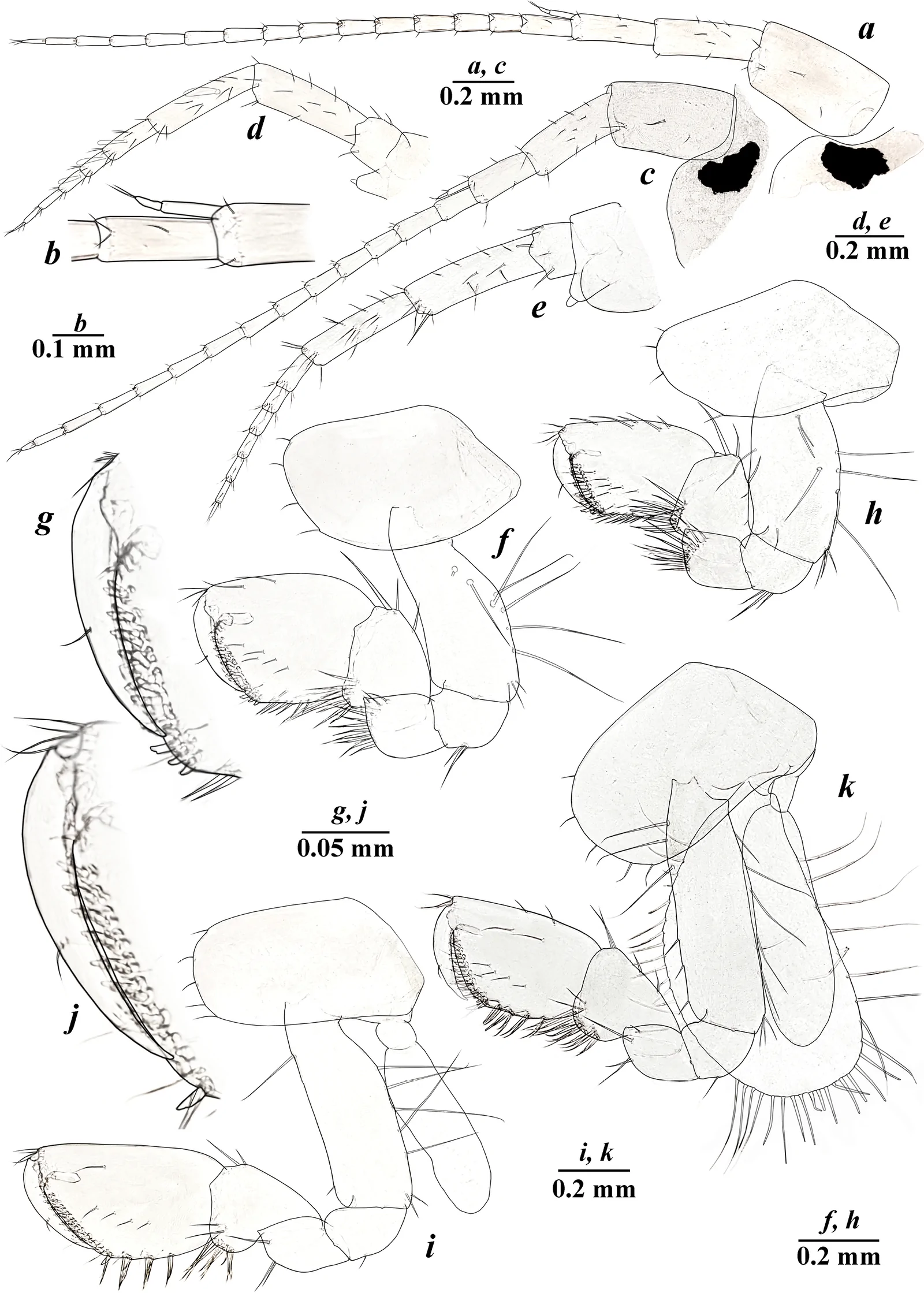
*Caucasirella lazareviensis* sp. nov. from Maryino, Psezuapse River, holotype ♂ (ZMMU Mb-1323) (**a, b, d, f, g, i, j**), paratype ♀ (ZMMU Mb-1324) (**c, e, h, k**): **a, c**. Antenna I; **b**. Accessory flagellum of AI; **d, e**. Antenna II; **f, h**. Gnathopod I; **g**. Palmar margin of propodus (palm) of gnathopod I; **i, k**. Gnathopod II; **j**. Palmar margin of propodus (palm) of gnathopod II.

***Head***: large, smooth, without rostrum, with subrounded lateral cephalic lobes and excavated anteroventral sinus; eyes (ommatidia) well seen (Fig. 9a, c); inter-antennal lobe wide, bluntly rounded anteriorly.

***Antenna I*** (Fig. 9a, c) ~ 45%–48% of body length, ~ 1.4' longer than antenna II; primary flagellum with 14–15 segments in ♂♂ and ♀♀, with aesthetascs on distal segments; accessory flagellum two-segmented, distal segment ~ 2' shorter than basal one.

***Antenna II*** (Fig. 9d, f): gland cone distinct, distally pointed; peduncle ~ 1.4' longer than flagellum, with robust setae tightly covering segments III and IV, peduncle of segment IV ~ 1.1' longer than segment V; flagellum six (♂♂) or seven (♀♀)-segmented; with calceoli on peduncle and flagellum in ♂♂ (Fig. 9e).

***Mandible***: left mandible incisor five-dentate, lacinia mobilis five-dentate, with four robust plumose accessory setae; molar process with one seta. Right mandible incisor four-dentate, lacinia mobilis trifurcate, both lobes with numerous protuberances; underlying with row of three robust plumose setae; molar process similar to left mandible. Palp three-segmented, segment II with six setae; segment III ~ 3.0' longer than wide, with straight margins, with eight or nine separate D-setae, two C-setae, one B-seta and three separate E-setae.

***Labrum*** (upper lip): oval, apical margin with numerous small fine setae.

***Labium*** (lower lip): inner lobes feebly developed.

***Maxilla I***: inner plate with three plumose marginal setae, outer plate with seven apical comb-spines; palp two-segmented, distal segment pubescent, ~ 1.8' of basal segment, apical margin of distal segment with seven simple setae.

***Maxilla II***: inner, outer plates covered in pubescent setae; outer plate smaller than inner plate, weakly narrowing distally, with 13 apical setae; inner plate narrowing slightly distally, with group of dense short setae on apex, with oblique row of five short plumose setae.

***Maxilliped***: inner plate much shorter than outer plate, with four spines and four simple setae apically and three spines subapically, outer plate narrow, with double row of 18 medial stiff setae of different lengths, palp quadriarticulate, article I with one seta on outer margin, article II with row of 18 simple setae on inner margin and one seta on outer margin, article III sub-quadrate; dactylus with one seta on outer margins, nail long with two thin setae at hinge.

***Gnathopod I*** (Fig. 9f, h): smaller than gnathopod II in ♂♂ and ♀♀: coxal plate sub-rectangular, distally rounded, with four apical and numerous facial setae, width/depth ratio ~ 0.70'; basis width/length ratio close to 0.42/1, with 0–1 setae on anterior margin, two or three long setae on inner face and four long setae on posterior margin; ischium approx. as long as wide, with tuft of posterodistal setae; merus quadrate, approx. as long as wide, with 10–13 distal setae; carpus ~ 0.52' of length of basis and ~ 0.62' of length of propodus, with three large serrated setae in inner margin and eight or nine serrated setae in outer margin; propodus swollen, ~ 1.3' longer than broad in ♂♂ (Fig. 9f) and ~ 1.4' longer than broad in ♀♀ (Fig. 9h), with 3–5 simple setae in anterior margin and 6–8 serrated setae in posterior margin; distal margin of palm oblique in ♂♂ and ♀♀ (Fig. 9g), with double row of six inner and six outer bifurcate robust setae (Fig. 9g); palm groove (depression) feebly developed, with five inner and four outer robust setae (Fig. 9g); dactylus with one outer seta.

***Gnathopod II*** (Fig. 9i, k): coxal plate sub-rectangular, with five or six apical and numerous facial setae, width/depth ratios ~ 0.6'; basis width/length ratio close to 0.31/1, with several (4–5) long setae inserted along posterior margin and 1–4 long and short simple setae in anterior margin; ischium approx. as long as wide, with tuft of posterodistal setae; merus quadrate, approx. as long as wide, with three or four distal setae; carpus ~ 0.48' of length of basis and ~ 0.62' of propodus, with 2–4 anterior and two or three groups of plumose posterior setae; propodus ~ 1.5' longer than broad in ♂♂ (Fig. 9i) and ~ 1.6' longer than broad in ♀♀ (Fig. 9k), with 0–2 simple anterior setae, one or two superior medial, four inferior medial and four or five groups of posterior setae; palm slightly oblique in ♂♂ and ♀♀ (Fig. 9j), with double row of seven inner and six outer bifurcate robust setae; palm groove (depression) feebly developed, with five inner and five outer robust setae (Fig. 9j); dactylus with 0–2 inner and one outer setae.

***Pereopod III***: similar in ♂♂ and ♀♀, coxal plate sub-rectangular, with five apical and numerous facial setae, width/depth ratio ~ 0.63'; basis ~ 3.9' as long as wide, with long anterior and posterior simple setae; ischium approx. as long as wide, with one distal seta; merus ~ 3.7' longer than wide, ~ 0.70' of basis, ~ 1.45' of carpus and mostly equal to propodus in length; carpus ~ 3.3' longer than wide, ~ 0.87' of propodus in length; propodus ~ 5.2' longer than wide, with five ventral spine-like setae; dactylus ~ 0.4' of propodus, relatively stout, with plumose seta on outer margin and two strong stiff setae on inner face.

***Pereopod IV***: similar in ♂♂ and ♀♀, coxal plate oval approx. as long as wide, with seven apical and numerous facial setae; basis ~ 4.1' as long as wide, with long anterior and posterior simple setae; ischium approx. as long as wide, with one distal seta; merus ~ 3.7' longer than wide, ~ 0.70' of basis, ~ 1.40' of carpus and mostly equal to propodus in length; carpus ~ 3.4' longer than wide, ~ 0.84' of propodus in length; propodus ~ 5.7' longer than wide, with five ventral spine-like setae; dactylus ~ 0.4' of propodus, relatively stout, with plumose seta on outer margin and two strong stiff setae on inner face.

***Pereopods V*–*VII***: length ratio of pereopods V/VI/VII close to 1/1.19/1.05 (♂♂) and 1/1.21/1.11 (♀♀).

***Pereopod V***: mostly similar in ♂♂ and ♀♀, coxal plate large, bilobate with distinct anterior and posterior lobes; anterior lobe with one margin simple seta, posterior lobe with one margin stout seta, each with numerous facial setae; basis ~ 1.52' as long as wide, with numerous facial setae, posterior margin slightly convex, armed with seven shallow serrations, with bluntly convex distal corner, anterior margin with six split-tipped robust and two distal setae; ischium approx. as long as wide, with three distal anterior setae; merus ~ 3.3' longer than wide, expanding posteriorly, ~ 0.72' of basis, ~ 0.94' of carpus and ~ 0.88' of propodus in length; carpus ~ 5.4' longer than wide, ~ 0.9' of propodus in length; propodus ~ 7.8' longer than wide, with four or five ventral spine-like setae; dactylus ~ 0.3' of propodus, with plumose seta on outer margin and three strong stiff setae on inner face.

***Pereopod VI***: mostly similar in ♂♂ and ♀♀, coxal plate bilobate, with distinct posterior and vestigial anterior lobes; anterior lobe without setae, posterior lobe with one margin stout seta, each with numerous facial setae; basis ~ 1.6' as long as wide, with numerous facial setae, posterior margin slightly convex, armed with seven shallow serrations, with bluntly convex distal corner, anterior margin with six split-tipped robust and 3 distal setae; ischium approx. as long as wide, with three distal anterior setae; merus ~ 3.3' longer than wide, expanding posteriorly, ~ 0.84' of basis and carpus and ~ 0.91' of propodus in length; carpus ~ 5.6' longer than wide, ~ 0.9' of propodus in length; propodus ~ 9.0' longer than wide, with six or seven ventral spine-like setae; dactylus ~ 0.3' of propodus, with plumose seta on outer margin and three strong stiff setae on inner face.

***Pereopod VII***. Male: coxal plate small, semi-lunar, with one posterior seta; basis ~ 1.55' as long as wide, with numerous facial setae, posterior margin convex, triangularly produced, armed with six shallow serrations, with bluntly convex distal corner, anterior margin with three split-tipped robust and three distal setae; ischium approx. as long as wide, with three distal anterior setae; merus ~ 2.9' longer than wide, expanding posteriorly, ~ 0.72' of basis and carpus and ~ 0.83' of propodus in length; carpus ~ 4.6' longer than wide, ~ 0.91' of propodus in length; propodus ~ 7.2' longer than wide, with three ventral spine-like setae; dactylus ~ 0.3' of propodus, with plumose seta on outer margin and three strong stiff setae on inner face. Female: coxal plate small, semi-lunar, with one posterior seta; basis ~ 1.57' as long as wide, with numerous facial setae, posterior margin significantly convex, armed with eight shallow serrations, with bluntly convex distal corner, anterior margin with five split-tipped robust and two distal setae; ischium approx. as long as wide, with three distal anterior setae; merus ~ 2.7' longer than wide, expanding posteriorly, ~ 0.6' of basis and carpus and ~ 0.83' of propodus in length; carpus ~ 4.7' longer than wide, ~ 0.92' of propodus in length; propodus ~ 6.3' longer than wide, with five ventral spine-like setae; dactylus ~ 0.3' of propodus, with plumose seta on outer margin and two strong stiff setae on inner face.

***Gills, brood plates***: coxal gills on somites II–VII, somites V–VII with lanceolate sternal gills. Male: coxal gills II–VII ovoid, gills/bases pereopod ratios ~ 0.83/1, 0.77/1, 0.76/1, 0.35/1, 0.30/1 and 0.24/1, respectively. Female: coxal gills II–VII ovoid, gills/bases pereopod ratios ~ 0.92/1, 0.97/1, 0.88/1, 0.45/1, 0.37/1 and 0.30/1, respectively. Slender, setaceous brood plates on somites II–V, decreasing in size posteriorly.

**Figure 10.**
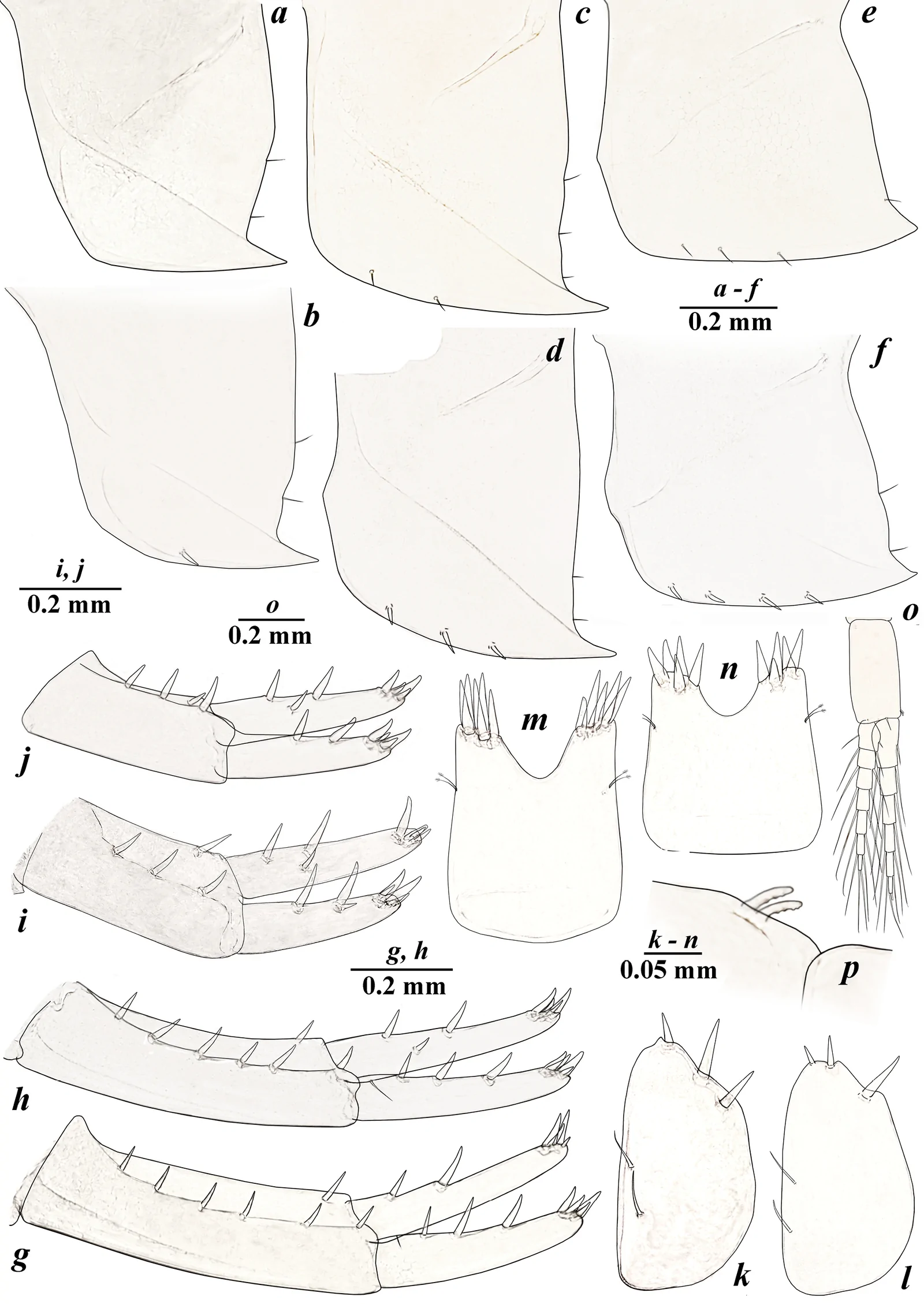
*Caucasirella lazareviensis* sp. nov. from Maryino, Psezuapse River, holotype ♂ (ZMMU Mb-1323) (**a, c, e, g, i, k, m, o, p**), paratype ♀ (ZMMU Mb-1324) (**b, d, f, h, j, l, n**): **a, b**. Epimeral plate I; **c, d**. Epimeral plate II; **e, f**. Epimeral plate III; **g, h**. Uropod I; **i, j**. Uropod II; **k, l**. Uropod III; **m, n**. Telson; **o**. Pleopod III; **p**. Coupling hooks of retinacula of pleopod III.

***Pleopods*** (Fig. 10o): peduncle with two coupling hooks in retinacules (Fig. 10p) of both ♂♂ and ♀♀; outer and inner rami with seven and six segments, respectively; basal segment of outer ramus without clothes-pin setae.

***Epimera***: Epimeral plate I (Fig. 10a, b) distally produced and strongly sharpened, ventral margin with 0–1 spine, posterior margin with two setae. Epimeral plate II (Fig. 10c, d) distally produced and strongly sharpened, ventral margin armed with two or three spines, posterior margin with two or three setae. Epimeral plate III (Fig. 10e, f) distally produced and strongly sharp, ventral margin armed with three or four spines, posterior margin with one or two setae.

***Urosomites*** completely fused, smooth.

***Uropod I*** (Fig. 10g, h) mostly similar in ♂♂ and ♀♀; peduncle ~ 3.3'–3.9' as long as wide (Fig. 10g); without distal marginally serrate process, with one dorso-internal robust spine, one subdistal spine and five dorso-external robust spines; exopodite mostly equal to endopodite; endopodite not paddle-like, with three dorsolateral and four apical spines; exopodite with three dorsolateral and four apical spines.

***Uropod II*** (Fig. 10i, j) mostly similar in ♂♂ and ♀♀; peduncle ~ 2.3' as long as wide, subequal in length to endopodite and exopodite, with three outer robust and one inner robust apical spines; exopodite slightly shorter than endopodite, with two outer and four apical robust spines; endopodite with two outer and four apical robust spines.

***Uropod III*** (Fig. 10k, l): vestigial, squamiform, slightly longer than telson, with three short spiniform setae apically and two setules on dorsal face.

***Telson*** (Fig. 10m, n): close to quadrate in shape, ~ 1.2' (♂♂) – 1.0' (♀♀) as long as broad; distal margin with relatively wide and deep V-shaped notch, ~ 0.23' of total length, each lobe armed with five robust spiniform setae, with two additional submarginal plumose setae.

##### Coloration.

The body and appendages yellowish or grayish transparent; well-pigmented black eyes well seen.

##### Size.

The largest collected ♂ has bl. 5.0 mm, ♀ – 5.5 mm.

##### GenBank accession numbers.

PZ956249, PZ956250.

##### Etymology.

The species is named after the Lazarevsky district of the Sochi Urban area, where it was discovered.

##### Taxonomic remarks.

From *C. shepsiensis* comb. nov. (after Sidorov 2015), the new species can be separated by 1) stouter propodus (palm) of gnathopod I, especially in ♂♂; 2) two coupling hooks in retinacules of pleopods (vs 3 hooks); 3) two or three ventral spines on epimeral plate II and three or four ventral spines on epimeral plate III (vs 5 spines and 5–6 spines, respectively); and 4) telson close to quadrate shape (vs rectangular, longer than wide).

From *C. mikhailovi* comb. nov. (see Marin and Palatov 2022), the new species can be separated by 1) stouter propodus (palm) of gnathopod I, especially in ♂♂; 2) pointing back posteroventral angle of epimeral plate I (vs pointing back and down); 3) two or three ventral spines on epimeral plate II and three or four ventral spines on epimeral plate III (vs 4 spines and always 3 spines, respectively); and 4) telson with relatively deeper notch.

From *C. fanagorica* comb. nov. (see Marin and Palatov 2022), the new species can be separated by 1) stouter propodus (palm) of gnathopod I, especially in ♂♂; 2) less oblique palmar margin of GII; 3) two coupling hooks in retinacules of pleopods (vs 3 hooks); and 4) relatively deeper notch on telson.

From *C. fontinalis* comb. nov. (see Marin and Palatov 2022), the new species can be separated by 1) longer propodus (palm) of gnathopod I, especially in ♀♀; 2) dactyli of ambulatory pereopods III–VII with two or three accessory ventral spines (vs 4 or 5 spines); 3) two coupling hooks in retinacules of pleopods (vs 3 hooks); and 4) relatively deeper notch on telson.

From *C. asheensis* comb. nov. (see Marin and Palatov 2022; present paper), the new species can be separated by 1) stouter propodus (palm) of gnathopod II, especially in ♂♂; 2) two coupling hooks in retinacules of pleopods (vs 3 hooks); and 3) relatively shallower notch on telson (vs notch deep, ~ 1/4–1/3 of telson length).

From *C. shapsuhica* sp. nov. (see above), the new species can be separated by 1) more spinulated segments of uropods I and II; 2) two coupling hooks in retinacules of pleopods; 3) uropod III with three distal spines (vs 4 spines); and 4) stouter and quadrate telson with relatively deep notch (vs rectangular telson, which is longer than wide).

For the differences from *C. mzymtiensis* sp. nov. see below.

##### Distribution and ecology.

Probably endemic hyporheic species, currently known only from a small river, being left tributary of the Psezuapse River (see above).

#### Caucasirella mzymtiensis sp. nov.

Taxon classificationAnimaliaAmphipodaCrangonyctidae

351C686F-EF08-52DC-8F0A-4ED42E59F831

https://zoobank.org/5905FD0F-A111-4916-9294-4FD8B2B2CBAE

Figs 11, 12

##### Material examined.

***Holotype***, • ♂ (bl. 4.5 mm) (ZMMU Mb-1325) – Russian Federation, Krasnodar Krai, Krasnodar Territory, Sochi city district, Adler, in a small spring (helocrene) in the valley of the Chvizhepse River, a right tributary of the Mzymta River, 43°38'28.65"N, 40°04'37.74"E, 230 m north of the border of the Chvizhepse village, ~ 272 m a.s.l., coll. D. Palatov, 14 April 2024. ***Paratype***. • ♀ (bl. 5.0 mm) (ZMMU Mb-1326) – same locality and data as holotype.

##### Other material.

• 10♂♂, 21♀♀ (LEMMI) – same locality and data as holotype.

##### Etymology.

The species is named after the Mzymta River, in the basin of which it was discovered. The Chvizhepse River, where the species was discovered, is its right tributary.

##### Description.

***Body***: moderately stout, stygomorphic.

***Head***: large, smooth, without rostrum, with subrounded lateral cephalic lobes and excavated anteroventral sinus; eyes (ommatidia) well seen; inter-antennal lobe wide, bluntly rounded anteriorly.

**Figure 11.**
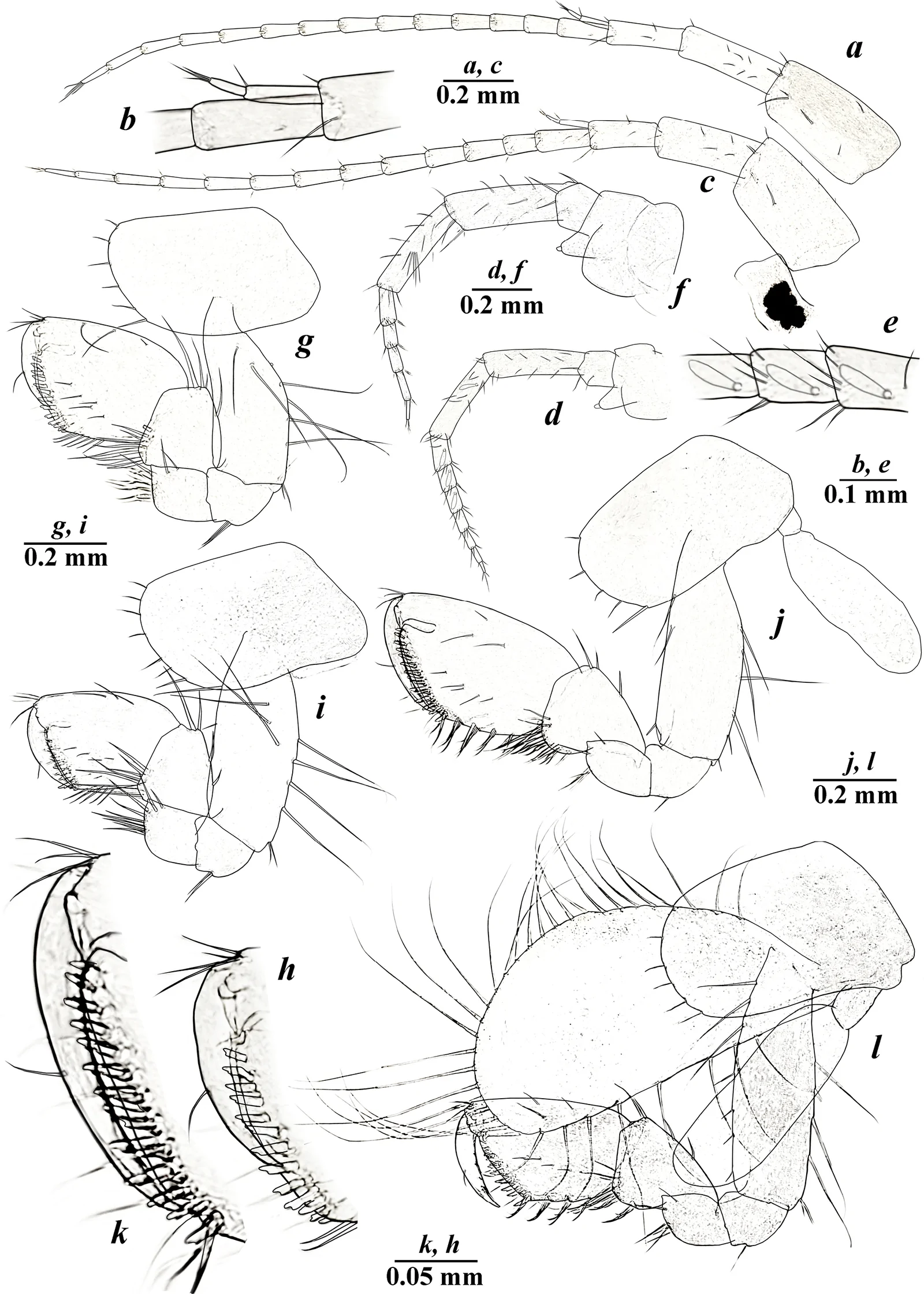
*Caucasirella mzymtiensis* sp. nov., holotype ♂ (ZMMU Mb-1325) (**a, b, d, e, g, h, j, k**), paratype ♀ (ZMMU Mb-1326) (**c, f, i, l**): **a, c**. Antenna I; **b**. Accessory flagellum of AI; **d, f**. Antenna II; **e**. Calceoli on flagellum of antenna II; **g, i**. Gnathopod I; **h**. Palmar margin of propodus (palm) of gnathopod I; **j, l**. Gnathopod II; **k**. Palmar margin of propodus (palm) of gnathopod II.

***Antenna I*** (Fig. 11a, c) ~ 45%–48% of body length, ~ 1.3'–1.4' longer than antenna II; primary flagellum with 13–14 segments in ♂♂ and ♀♀, with aesthetascs on distal segments; accessory flagellum two-segmented, distal segment ~ 2' shorter than basal one.

***Antenna II*** (Fig. 11d, f): gland cone distinct, distally pointed; peduncle ~ 1.4' longer than flagellum, with robust setae tightly covering segments III and IV, peduncle of segment VI ~ 1.1' longer than segment V; flagellum seven (♂♂) or five (♀♀)-segmented; with calceoli on peduncle and flagellum in ♂♂ (Fig. 11e).

***Mandible***: left mandible incisor five-dentate, lacinia mobilis five-dentate, with three robust plumose accessory setae; molar process with one seta. Right mandible incisor four-dentate, lacinia mobilis bifurcate, both lobes with numerous protuberances; underlying with row of three robust plumose setae; molar process similar to left mandible. Palp three-segmented, segment II with five setae; segment III ~ 3.0' longer than wide, with straight margins, with five or six separate D-setae, two C-setae, one B-seta and three separate E-setae.

***Labrum*** (upper lip): oval, apical margin with numerous small fine setae.

***Labium*** (lower lip): inner lobes feebly developed.

***Maxilla I***: inner plate with three plumose marginal setae, outer plate with seven apical comb-spines; palp two-segmented, distal segment pubescent, ~ 1.7' of basal segment, apical margin of distal segment with seven simple setae.

***Maxilla II***: inner, outer plates covered in pubescent setae; outer plate smaller than inner plate, weakly narrowing distally, with 13 apical setae; inner plate narrowing slightly distally, with group of dense short setae on apex, with oblique row of five short plumose setae.

***Maxilliped***: inner plate much shorter than outer plate, with four spines and three simple setae apically and two setae subapically, outer plate narrow, with double row of 14 medial stiff setae of different lengths, palp quadriarticulate, article I with one seta on outer margin, article II with row of 17 simple setae on inner margin and one seta on outer margin, article III sub-quadrate; dactylus with one seta on outer margins, nail long with two thin setae at hinge.

***Gnathopod I*** (Fig. 11g, i): smaller than gnathopod II in ♂♂ and ♀♀: coxal plate sub-rectangular, distally rounded, with six apical and numerous facial setae, width/depth ratio ~ 0.66'; basis width/length ratio close to 0.4/1, with 0–1 setae on anterior margin, one or two long setae on inner face and four or five long setae on posterior margin; ischium approx. as long as wide, with tuft of posterodistal setae; merus quadrate, approx. as long as wide, with nine or ten distal setae; carpus ~ 0.5' of length of basis and ~ 0.56' of length of propodus, with nine serrated setae in inner margin and four serrated setae in outer margin; propodus swollen, ~ 1.4' longer than broad in ♂♂ (Fig. 11g) and ~ 1.5' longer than broad in ♀♀ (Fig. 11i), with three or four simple setae in anterior margin and five or six serrated setae in posterior margin; distal margin of palm slightly oblique in ♂♂ and ♀♀ (Fig. 11h), with double row of six inner and seven outer bifurcate robust setae (Fig. 11h); palm groove (depression) feebly developed, with three inner and three outer robust setae; dactylus with one outer seta.

***Gnathopod II*** (Fig. 11j, l): coxal plate sub-rectangular, with five apical and numerous facial setae, width/depth ratios ~ 0.4'; basis width/length ratio close to 0.35/1, with several (4 or 5) long setae inserted along posterior margin and 2–6 short and long simple setae in anterior margin; ischium approx. as long as wide, with tuft of posterodistal setae; merus quadrate, approx. as long as wide, with five or six distal setae; carpus ~ 0.52' of length of basis and ~ 0.58' of propodus, with two or three anterior and two or three groups of plumose posterior setae; propodus ~ 1.6' longer than broad in ♂♂ (Fig. 11j) and ~ 1.7' longer than broad in ♀♀ (Fig. 11l), with one or two simple anterior setae, one or two superior medial, four or five inferior medial and four or five groups of posterior setae; palm slightly oblique in ♂♂ and ♀♀ (Fig. 11k), with double row of six inner and seven outer bifurcate robust setae; palm groove (depression) feebly developed, with three inner and two outer robust setae (Fig. 11k); dactylus with 0–2 inner and one outer setae.

***Pereopod III***: similar in ♂♂ and ♀♀, coxal plate sub-rectangular, with six apical and numerous facial setae, width/depth ratio ~ 0.52'; basis ~ 3.7' as long as wide, with long anterior and posterior simple setae; ischium approx. as long as wide, with one distal seta; merus ~ 3.4' longer than wide, ~ 0.68' of basis, ~ 1.29' of carpus and mostly equal to propodus in length; carpus ~ 3.7' longer than wide, ~ 0.87' of propodus in length; propodus ~ 5.6' longer than wide, with five ventral spine-like setae; dactylus ~ 0.4' of propodus, relatively stout, with plumose seta on outer margin and two strong stiff setae on inner face.

***Pereopod IV***: similar in ♂♂ and ♀♀, coxal plate oval approx. as long as wide, with nine apical and numerous facial setae; basis ~ 3.9' as long as wide, with long anterior and posterior simple setae; ischium approx. as long as wide, with one distal seta; merus ~ 3.5' longer than wide, ~ 0.72' of basis, ~ 1.36' of carpus and mostly equal to propodus in length; carpus ~ 3.6' longer than wide, ~ 0.82' of propodus in length; propodus ~ 6.0' longer than wide, with five ventral spine-like setae; dactylus ~ 0.4' of propodus, relatively stout, with plumose seta on outer margin and three strong stiff setae on inner face.

***Pereopods V*–*VII***: length ratio of pereopods V/VI/VII close to 1/1.21/1.065 (♂♂) and 1/1.22/1.07 (♀♀).

***Pereopod V***: mostly similar in ♂♂ and ♀♀, coxal plate large, bilobate with distinct anterior and posterior lobes; anterior lobe with one margin simple seta, posterior lobe with one margin stout seta, each with numerous facial setae; basis ~ 1.47' as long as wide, with numerous facial setae, posterior margin slightly convex, armed with eight shallow serrations, with bluntly convex distal corner, anterior margin with seven split-tipped robust and two distal setae; ischium approx. as long as wide, with three distal anterior setae; merus ~ 2.9' longer than wide, expanding posteriorly, ~ 0.74' of basis, ~ 0.94' of carpus and ~ 0.96' of propodus in length; carpus ~ 5.4' longer than wide, ~ 0.9' of propodus in length; propodus ~ 8.2' longer than wide, with four or five ventral spine-like setae; dactylus ~ 0.3' of propodus, with plumose seta on outer margin and three strong stiff setae on inner face.

***Pereopod VI***: mostly similar in ♂♂ and ♀♀, coxal plate bilobate, with distinct posterior and vestigial anterior lobes; anterior lobe without setae, posterior lobe with one margin stout seta, each with numerous facial setae; basis ~ 1.60' as long as wide, with numerous facial setae, posterior margin slightly convex, armed with eight shallow serrations, with bluntly convex distal corner, anterior margin with six split-tipped robust and three distal setae; ischium approx. as long as wide, with two distal anterior setae; merus ~ 3.4' longer than wide, expanding posteriorly, ~ 0.77' of basis and carpus and ~ 0.81' of propodus in length; carpus ~ 6.0' longer than wide, ~ 0.93' of propodus in length; propodus ~ 9.1' longer than wide, with four ventral spine-like setae; dactylus ~ 0.3' of propodus, with plumose seta on outer margin and three strong stiff setae on inner face.

***Pereopod VII***. Male: coxal plate small, semi-lunar, with one posterior seta; basis ~ 1.70' as long as wide, with numerous facial setae, posterior margin convex, triangularly produced, armed with nine shallow serrations, with bluntly convex distal corner, anterior margin with seven split-tipped robust and 3 distal setae; ischium approx. as long as wide, with two distal anterior setae; merus ~ 2.9' longer than wide, expanding posteriorly, ~ 0.64' of basis and carpus and ~ 0.74' of propodus in length; carpus ~ 5.2' longer than wide, ~ 0.86' of propodus in length; propodus ~ 7.8' longer than wide, with five ventral spine-like setae; dactylus ~ 0.3' of propodus, with plumose seta on outer margin and three strong stiff setae on inner face. Female: coxal plate small, semi-lunar, with one posterior seta; basis ~ 1.7' as long as wide, with numerous facial setae, posterior margin significantly convex, armed with ten shallow serrations, with bluntly convex distal corner, anterior margin with six split-tipped robust and three distal setae; ischium approx. as long as wide, with two distal anterior setae; merus ~ 2.6' longer than wide, expanding posteriorly, ~ 0.57' of basis and carpus and ~ 0.84' of propodus in length; carpus ~ 4.1' longer than wide, ~ 0.9' of propodus in length; propodus ~ 5.4' longer than wide, with three ventral spine-like setae; dactylus ~ 0.3' of propodus, with plumose seta on outer margin and two strong stiff setae on inner face.

***Gills, brood plates***: coxal gills on somites II–VII, somites V–VII with lanceolate sternal gills. Male: coxal gills II–VII ovoid, gills/bases pereopod ratios ~ 0.85/1, 0.77/1, 0.76/1, 0.47/1, 0.30/1 and 0.22/1, respectively. Female: coxal gills II–VII ovoid, gills/bases pereopod ratios ~ 0.93/1, 0.90/1, 0.92/1, 0.48/1, 0.27/1 and 0.27/1, respectively. Slender, setaceous brood plates on somites II–V, decreasing in size posteriorly.

**Figure 12.**
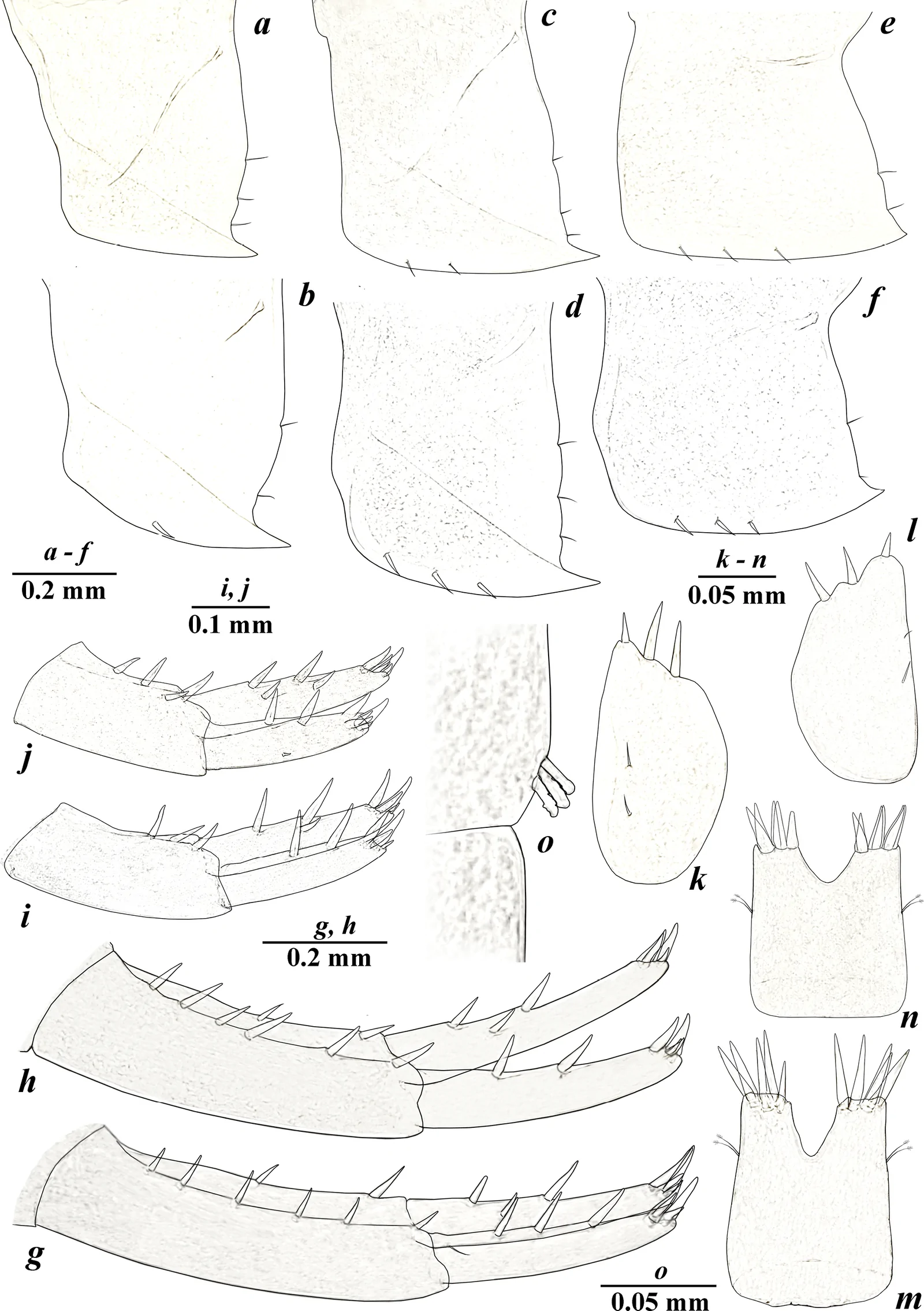
*Caucasirella mzymtiensis* sp. nov., holotype ♂ (ZMMU Mb-1325) (**a, c, e, g, i, k, m, o**), paratype ♀ (ZMMU Mb-1326) (**b, d, f, h, j, l, n**): **a, b**. Epimeral plate I; **c, d**. Epimeral plate II; **e, f**. Epimeral plate III; **g, h**. Uropod I; **i, j**. Uropod II; **k, l**. Uropod III; **m, n**. Telson; **o**. Coupling hooks of retinacula of pleopod III.

***Pleopods***: peduncle with three coupling hooks in retinacules (Fig. 12o) of both ♂♂ and ♀♀; outer and inner rami with six and six segments, respectively; basal segment of outer ramus without clothes-pin setae.

***Epimera***: Epimeral plate I (Fig. 12a, b) distally produced and strongly sharpened, ventral margin with 0–1 spine, posterior margin with two or three setae. Epimeral plate II (Fig. 12c, d) distally produced and strongly sharpened, ventral margin armed with two or three spines, posterior margin with three setae. Epimeral plate III (Fig. 12e, f) distally produced and strongly sharpened, ventral margin armed with three spines, posterior margin with two setae.

***Urosomites*** completely fused, smooth.

***Uropod I*** (Fig. 12g, h) mostly similar in ♂♂ and ♀♀; peduncle ~ 3.5'–3.8' as long as wide, without distal marginally serrate process, with one dorso-internal robust spine, one subdistal spine and six dorso-external robust spines; exopodite mostly equal to endopodite; endopodite not paddle-like, with two or three dorsolateral and four apical spines; exopodite with two or three dorsolateral and four apical spines.

***Uropod II*** (Fig. 12i, j) mostly similar in ♂♂ and ♀♀; peduncle ~ 2.5' as long as wide, subequal in length to endopodite and exopodite, with two outer, one inner and one or two subdistal robust spines; exopodite slightly shorter than endopodite, with two outer and four apical robust spines; endopodite with two outer and four apical robust spines.

***Uropod III*** (Fig. 12k, l): vestigial, squamiform, slightly longer than telson, with three short spiniform setae apically and two setules on dorsal face.

***Telson*** (Fig. 12m, n): close to quadrate in shape, ~ 1.25' (♂♂) – 1.10' (♀♀) as long as broad; distal margin with relatively wide and deep U-shaped notch, ~ 0.25–0.30' of total length, each lobe armed with five or six robust spiniform setae, with two additional submarginal plumose setae.

##### Coloration.

The body and appendages yellowish or grayish transparent; well-pigmented black eyes well seen.

##### Size.

The largest collected ♂ has bl. 5.0 mm, ♀ – 5.5 mm.

##### GenBank accession numbers.

PZ956247, PZ956248.

##### Taxonomic remarks.

The species is closely related to *Caucasirella lazareviensis* sp. nov. (see above) differing from it by 1) more spinulated segments of uropods I and II; 2) three coupling hooks in retinacules of pleopods (vs 2 hooks); 3) epimeral plate III with three ventral spines (vs always 3 or 4 spines); 4) telson with relatively deeper notch.

From other Caucasian *Caucasirella* gen. nov. the new species can be separated mostly by the morphological features, similar to *Caucasirella lazareviensis* sp. nov. From *Caucasirella shepsiensis* comb. nov. (after Sidorov, 2015), the new species can be separated by 1) stouter propodus (palm) of gnathopod I, especially in ♂♂; 2) two or three ventral spines on epimeral plate II and three or four ventral spines on epimeral plate III (vs 5 spines and 5 or 6 spines, respectively); and 3) telson close to quadrate shape (vs rectangular, longer than wide).

From *C. mikhailovi* comb. nov. (see Marin and Palatov 2022), the new species can be separated by 1) stouter propodus (palm) of gnathopod I, especially in ♂♂; 2) three coupling hooks in retinacules of pleopods (vs 1 or 2 hooks); 3) pointing back posteroventral angle of epimeral plate I (vs pointing back and down); 4) two or three ventral spines on epimeral plate II and three or four ventral spines on epimeral plate III (vs 4 spines and always 3 spines, respectively); and 5) telson with relatively deeper notch.

From *C. fanagorica* comb. nov. (see Marin and Palatov 2022), the new species can be separated by 1) stouter propodus (palm) of gnathopod I, especially in ♂♂; 2) less oblique palmar margin of gnathopod II; and 3) relatively deeper notch on telson.

From *C. fontinalis* comb. nov. (see Marin and Palatov 2022), the new species can be separated by 1) longer propodus (palm) of gnathopod I, especially in ♀♀; 2) dactyli of ambulatory pereopods III–VII with two or three accessory ventral spines (vs 4 or 5 spines); and 3) relatively deeper notch on telson.

From *C. asheensis* comb. nov. (see Marin and Palatov 2022; present paper), the new species can be separated by 1) stouter propodus (palm) of gnathopod II, especially in ♂♂; and 2) relatively shallower notch on telson (vs notch deep, ~ 1/4–1/3 of telson length).

From *C. shapsuhica* sp. nov. (see above), the new species can be separated by 1) more spinulated segments of uropods I and II; 2) uropod III with 3 distal spines (vs 4 spines); and 3) stouter and quadrate telson with relatively deep notch (vs rectangular telson, which is longer than wide).

##### Distribution and ecology.

Probably endemic riverine hyporheic species, currently known only from a small spring (helocrene) in the valley of the Chvizhepse River (see above).

##### Description of juveniles of the representatives of the genus *Caucasirella* gen. nov., based on *C. lazareviensis* sp. nov.

Lack of knowledge about morphological variations during the growth of amphipod crustaceans can lead to misidentification of species (Violante-Huerta et al. 2021). Juveniles of the newly described species *C. lazareviensis* sp. nov. were studied for the first time, providing the first insight into the larval morphology of the genus *Caucasirella* gen. nov. In fact, morphological variations have not been described for any crangonyctid species before. However, juveniles differ significantly from adults, with features in the morphology of their dactyli, ambulatory pereopods, telson, and uropod III, as well as the number of coupling hooks in their pleopod retinacula. These observations enrich the description of *C. lazareviensis* sp. nov. at the early stages of its growth and support the need for further taxonomic studies to help identify species at different developmental stages.

##### Brief description

**Figure 13.**
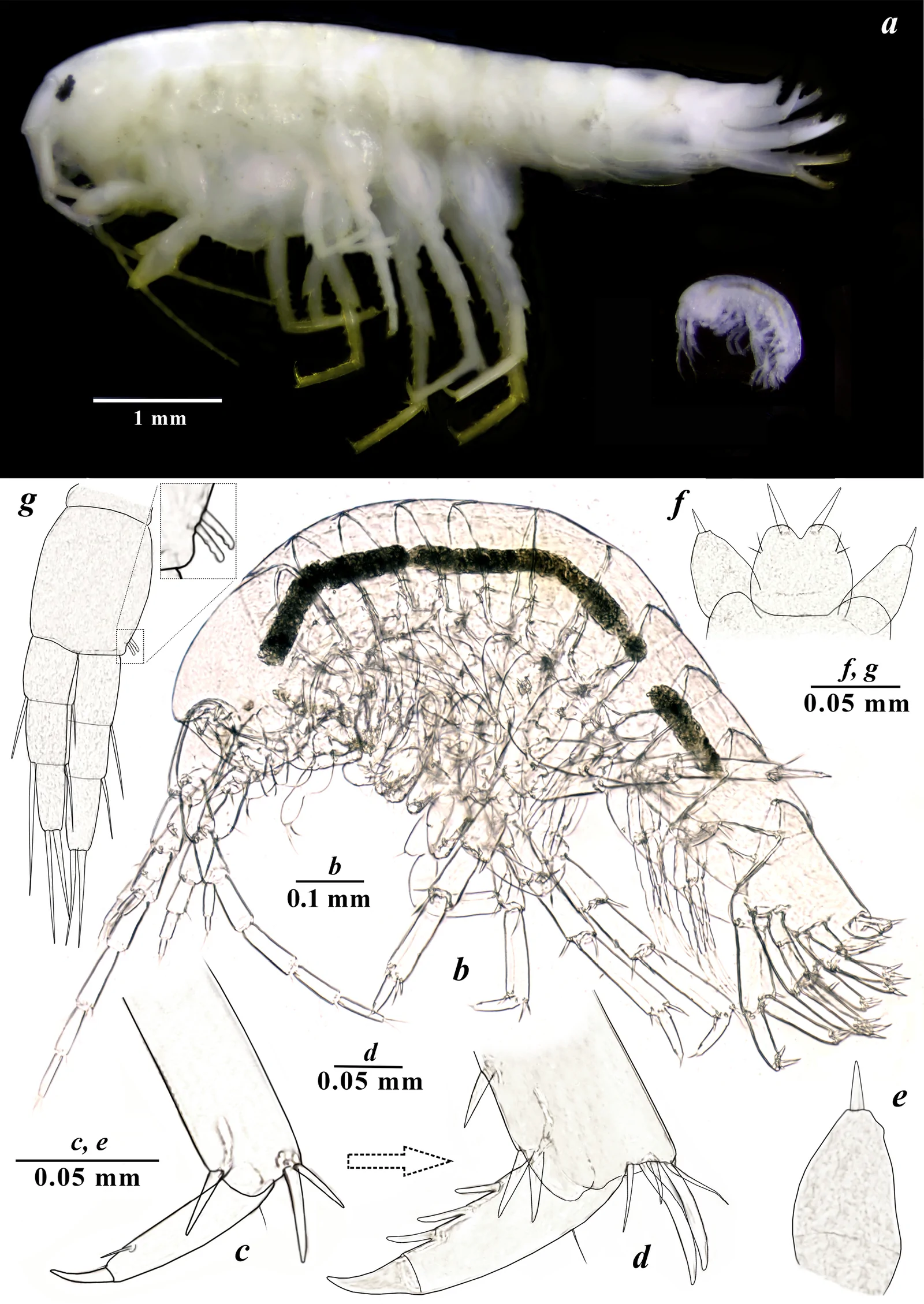
*Caucasirella lazareviensis* sp. nov. from Maryino, Psezuapse River, fixed mature ♀ (**a, d**) and its off-spring (juvenile) (**b, c, e–g**): **a**. General lateral view; **b**. General lateral view; **c, d**. Dactylus of PIII; **e**. Uropod III, **f**. Telson and uropods III; **g**. Pleopod III and coupling hooks (enlarged).

(Fig. 13). ***Body***: in general view similar to adults, moderately stout, stygomorphic, transparent (see Fig. 13a, b); Total length ~ 1.0–1.2 mm. ***Head***: smooth, eyes (ommatidia) not seen (Fig. 13b). ***Antenna I*** (Fig. 13a, c) ~ 50% of body length, ~ 2' longer than antenna II; primary flagellum with 4 segments; accessory flagellum two-segmented, distal segment ~ 2' shorter than basal one. ***Antenna II*** (Fig. 13b) with three-segmented flagellum. ***Pereopod III*** with stout mostly smooth segments, propodus ~ 3' longer than wide, with only most distal one spine-like seta (Fig. 13c); dactylus ~ 0.4' of propodus, ~ 4' as long as wide, with plumose seta on outer margin, without strong stiff setae on inner face (which are well developed in adults (Fig. 13d)), with one small subdistal simple seta. ***Pereopod IV*–*VII*** mostly similar to PIII. ***Pleopods***: with stout peduncle with two coupling hooks in retinacules (Fig. 13g); outer and inner rami with three segments. ***Epimera*** unarmed. ***Urosomites*** completely fused, smooth. ***Uropods I and II*** with smooth segments, armed with 2–4 subdistal spines only. ***Uropod III*** (Fig. 13e, f): vestigial, squamiform, equal to telson, with only one short spiniform seta apically (Fig. 13e). ***Telson*** (Fig. 13f): close to quadrate in shape, approx. as long as broad; distal margin with shallow U-shaped notch, ~ 0.15–0.20' of total length, each lobe armed with one robust spiniform seta, with two additional submarginal plumose setae.

##### ZooBank taxon ID.

The electronic version of this article in Portable Document Format will represent a published work according to the International Commission on Zoological Nomenclature (ICZN), and hence the new names contained in the electronic version are effectively published under that Code from the electronic edition alone. This published work and the nomenclatural acts it contains have been registered in ZooBank, the online registration system for the ICZN. The ZooBank Life Science Identifiers (LSID) for this publication is: https://zoobank.org/65BE68F8-869C-443C-8166-AF38D9EA3DE2. The online version of this work is archived and available from the following digital repositories: ZooKeys, PubMed Central, CLOCKSS and others. The LSID for the newly described species, for *Caucasirella* Marin & Palatov gen. nov. is https://zoobank.org/64E594C3-33E5-4EB9-AF2C-DA623633979F, and newly described species *Caucasirella shapsuhica* sp. nov. is https://zoobank.org/1221156A-75B2-4A69-8C7D-10E6D1DAA955, *Caucasirella lazareviensis* sp. nov. is https://zoobank.org/E9C108AA-3217-4F25-B01B-7BBA993FE07F and *Caucasirella mzymtiensis* sp. nov. is https://zoobank.org/5905FD0F-A111-4916-9294-4FD8B2B2CBAE.

## Discussion

**Figure 14.**
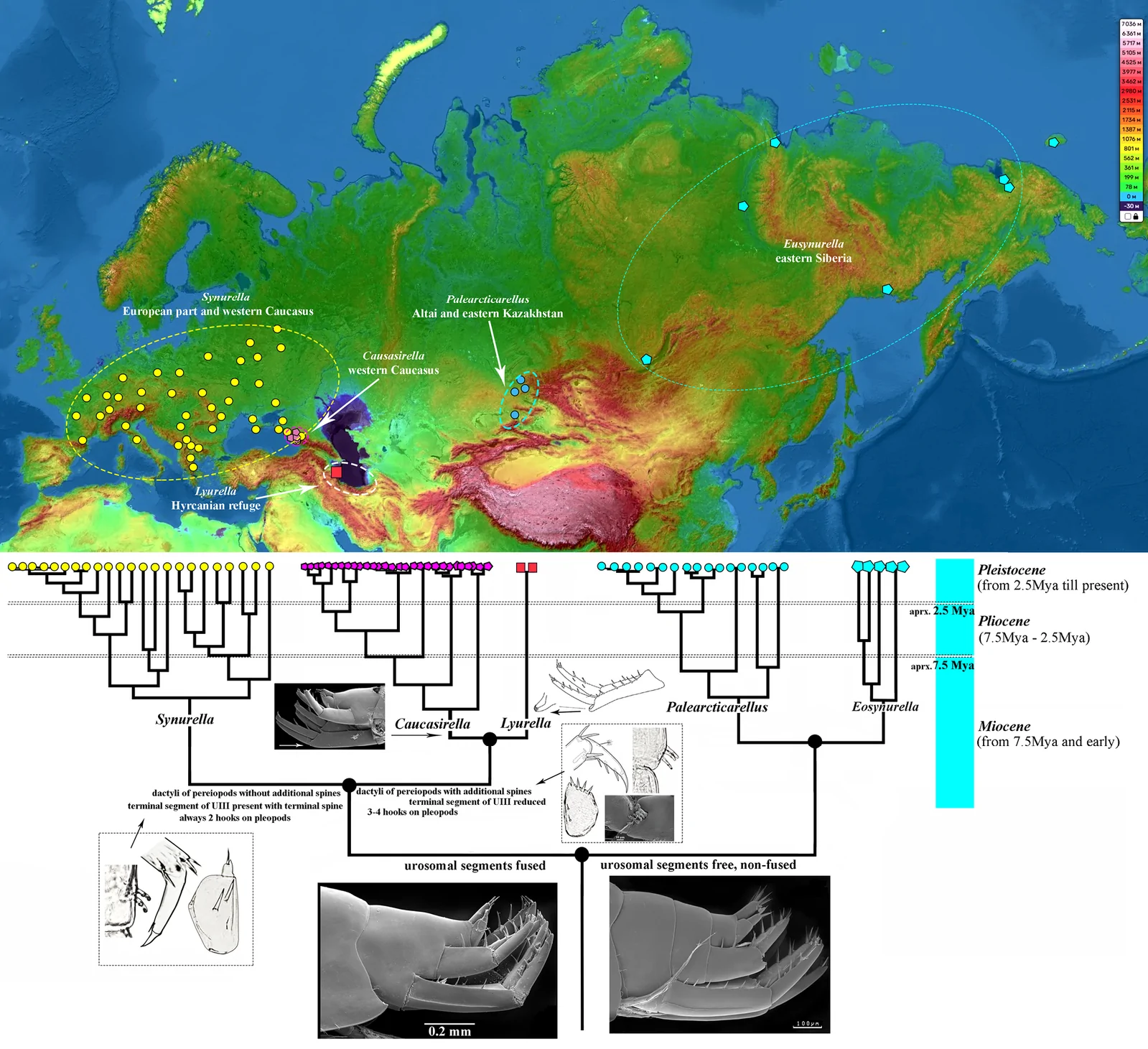
Distribution map of the species of the genera referring to main “*Synurella*” clade of the family Crangonyctidae combined with morphology-based relationships (tree) between the recently described genera, *Synurella* (yellow circle), *Caucasirella* gen. nov. (violet square), *Lyurella* (*L. hyrcana*) (red square), *Palearcticarellus* (turquoise circle) and *Eosynurella* (blue circle)). The main characteristic apomorphic morphological features are indicated for branches. The proposed time of divergence corresponds to the species-level divergence times within genera calculated within time-calibrated phylogenetic reconstructions (MCMC) for each genus, shown in Fig. 1 (see Discussion for more details).

Recent molecular genetic analyses have revolutionized the taxonomy of Palearctic Crangonyctidae, prompting significant revisions. New genera have been erected (e.g., Palatov and Marin 2020; Marin and Palatov 2021a), while others have been resurrected (see Marin and Palatov 2023b). This article continues a series of taxonomic studies and offers a solution for classifying the known genera within the main branches (clades) of the family. Although further taxonomic changes are still possible, molecular genetic (uni-locus COI mtDNA based phylogeny) (Fig. 1), known morphological features, and geographical distribution (Fig. 14) support the recognition of a new genus, *Caucasirella* gen. nov., for the western Caucasian species previously assigned to the genus *Lyurella* (see above). Furthermore, the pronounced phylogenetic divergence between *Lyurella* and *Caucasirella* gen. nov. strongly suggests their ancient, relict origin (see Fig. 1; also see below). Based on COI mtDNA substitution rates, the estimated time of divergence between these two genera is ca 10–7.5 Mya, placing this crucial evolutionary event within the Miocene epoch (see Fig. 1). We further assert that the current known diversity of Palearctic crangonyctids, and particularly within *Caucasirella* gen. nov. and *Lyurella*, remains underestimated.

All recently known and genetically confirmed representatives of the “*Synurella*” clade (after Marin and Palatov 2021a), including *Synurella* (Wrześniowski, 1877), *Eosynurella* (Martynov, 1931), *Lyurella* (Derzhavin, 1939), *Palearcticarellus* (Palatov & Marin, 2020) and *Caucasirella* gen. nov., have been found in the northern Palearctic, extending from Central Europe through the Altai and Baikal regions to Chukotka and Wrangel Island (see Fig. 14). Probably, representatives of the clade also inhabit the Beringian sector of the Nearctic (Hultén 1937; Hopkins 1967; Hoffecker and Elias 2007). However, the only Nearctic species, Synurella (Eosynurella) johanseni Shoemaker, 1920 (after Martynov 1931), described from tundra lakes and swamps around the city of Teller, Alaska (USA) (Shoemaker 1920), has not yet been subjected to molecular genetic analysis to verify its generic status. By way of discussion, we propose that the current distribution of these genera is “phylogenetically congruent”, reflecting the evolutionary branching order and aligning well with gradual stages of allopatric divergence (vicariance) within the group (see Fig. 14). If this scenario holds true, it suggests that the historical process of divergence and specialization was driven more by the temporary geographic isolation, and subsequent environmental pressures, rather than by extensive dispersal (with some exceptions, see below). This pattern aligns with classic examples of relic faunas (Habel et al. 2010; Hájek et al. 2011; Grandcolas et al. 2014), representing a key interpretative outcome of these data (see Fig. 14).

The origin of the “*Synurella*” clade has not been reliably clarified. It is estimated that this clade split from other lineages of the family Crangonyctidae from ca 55–60 Mya (see Copilaş -Ciocianu et al. 2019) to ca 5–39 Mya, with the mean estimation of split ca 12 Mya (Miocene) (see Palatov and Marin 2021b; present paper). Our reconstructions (Fig. 14) implicate the ancient Paratethys Sea (66–12 Mya) (Popov et al. 2004, 2006) in its origin and early evolution. Based on our reconstructions (see Fig. 14), It is suggested that this huge water basin maximum extended “from Paris to Altai” ca 11 Mya (Caves et al. 2017; Li et al. 2020; Popov and Patina 2023). A key observation is that the non-overlapping modern distributions of genera within the “*Synurella*” clade (see Fig. 14) occur within regions historically associated with the Paratethys (Popov et al. 2004, 2006). The presence of the genus *Palearcticarellus* in Altai may be explained by the Late Miocene eastward expansion of Paratethys into the Lake Zaysan area (Tarbagatay District, East Kazakhstan) and adjacent lower Russian Altai foothills (Caves et al. 2017; Li et al. 2020). We propose that the gradual Pliocene regression of Paratethys (Popov et al. 2004, 2006; Marin and Palatov 2022; Popov and Patina 2023), coupled with rapid intra-generic diversification in the last 8–5 Mya and a partial transition to hypogean lifestyles (troglomorphism) (Palatov and Marin 2020, 2021b; Marin and Palatov 2021b, 2022), provides a compelling explanation for the current distribution (excluding *Eosynurella*) (see Fig. 14).

The distribution of *Eosynurella* across northern Eurasia is likely a result of secondary dispersal. These predominantly epigean species inhabit tundra swamps and major Siberian/Far Eastern River basins (Martynov 1931; Marin and Palatov 2023). Their long-distance dispersal could have occurred via extensive river systems during flood events (e.g., Trontelj et al. 2009; Van Leeuwen et al. 2013; Copilaș-Ciocianu et al. 2017, 2018) or through interconnected tundra biotopes. During the Pleistocene, radical alterations to northern Eurasian River systems by glaciations (2.58 Mya–11.7 Tya), including the formation of massive glacial lakes like the West Siberian Glacial Lake (Mangerud et al. 2001; Krinner et al. 2004; Sheinkman et al. 2022), created different dispersal pathways. Alternatively, passive transport by large wading birds, similar to hypotheses for *Synurella* (Marin and Palatov 2022), *Gammarus* (Gammaridae) (Coughlan et al. 2017; Sket et al. 2019), *Niphargus* Schiödte, 1849 (Niphargidae) (Palatov and Marin 2021a; Morhun et al. 2022) or other small crustaceans (e.g., Marin and Statkevich 2025), is also a plausible mechanism for *Eosynurella* dispersal.

The current geographical distribution of the genera *Lyurella* and *Caucasirella* gen. nov. is best explained by the combined effects of Paratethys reduction and the uplift in the Caucasus Mountain ranges, a process that commenced ca 25 Mya (Milanovsky and Khain 1963) and was mostly active since 10–5 Mya (Popkov 2006; Vasey et al. 2020; Mossar et al. 2022; Vasey et al. 2023; Kuznetsov and Romanyuk 2025). Currently, *Lyurella* (*L. hyrcana* and probably several undescribed species) is found in the eastern part of the Caucasus, SE Azerbaijan and northern Caspian coast of Iran within the Hyrcanian refuge (Derzhavin 1939; Ruffo 1974; Aliev 2000; Sidorov 2015). At the same time, *Synurella* and *Caucasirella* gen. nov. inhabit areas from Europe eastward to the northern boundaries of the Colchida Lowland of the Eastern Black Sea (Colchis refugium), with the easternmost species (e.g., *Synurella ispani* Palatov & Marin, 2021 and *Synurella spiridonovi* Marin & Palatov, 2021) known from the coastal swamps of Poti and Kobuleti (Palatov and Marin 2021b). Crucially, there are no recent records of these genera in the intervening central Georgia, along the southern slope of the western Caucasus, or west of Krasnodar Krai along its northern slope (see Fig. 14). This gap suggests that the central and western Caucasus Range functions as a significant biogeographic barrier, effectively separating the western distribution of *Synurella* + *Caucasirella* gen. nov. from the eastern distribution of *Lyurella* (see Fig. 14). Nevertheless, the presence of other Crangonyctidae, such as *Diasynurella wachuschtii* (Behning, 1940) from the mountainous Samtskhe-Javakheti region, on the Tskhratskaro pass, near Bakuriani (central Georgia) (Behning 1940; Palatov and Marin 2023), indicates that this region is biologically rich and that further discoveries within the “*Synurella*” clade are plausible.

We would like also to discuss here the key morphological features that define the principal main branches of the “*Synurella*” clade (see Fig. 14). Integrative studies combining molecular and morphological studies (see Fig. 14) delineate two clades: “*Synurella* + *Caucasirella* + *Lyurella*”, and “*Palearcticarellus* + *Eosynurella*”. The most reliable feature that separates these groups, even at present, is the state of urosomal segment fusion. In all known representatives of the “*Synurella* + *Caucasirella* + *Lyurella*” clade, the urosome represents a single unit with completely fused segments (Karaman 1974; Holsinger 1977; Sidorov 2015; Marin and Palatov 2021), a feature evident even for juveniles of *Caucasirella* gen. nov. (see Fig. 13). In contrast, urosomal segments in the “*Palearcticarellus* + *Eosynurella*” clade are always separated (Martynov 1931; Palatov and Marin 2020). We believe that urosomal segmentation represents a significant, basal divergence and a key specialization event between these evolutionary lineages. Most likely ancestral condition for crangonyctids is the presence of separate urosomal segments as it is currently represented in most taxa (e.g., Holsinger 1977).

Analyzing the juvenile morphology of *C. lazareviensis* sp. nov. (Fig. 13) leads us to identify two basal autapomorphies for the ”*Synurella* + *Caucasirella* gen. nov. + *Lyurella*” clade (Fig. 14): the ancestral condition of dactyli of ambulatory pereopods without inner spines and only two pleopodal retinacular coupling hooks. While adults of *Synurella* maintain this ancestral state (Karaman 1974; Holsinger 1977; Palatov and Marin 2021b; Marin and Palatov 2022b, 2025), *L. hyrcana* and all species of *Caucasirella* gen. nov. have dactyli with additional spines in adult, and the pleopodal retinacula with 5 coupling hooks (Sidorov 2015; Marin and Palatov 2021b).

Further evolutionary differentiation distinguishes *Lyurella* from *Caucasirella* gen. nov. A notable difference lies in the morphology of peduncle of uropod I in ♂♂: *Lyurella* has a well-developed peduncular distoventral serrate process (Sidorov 2015), while it is almost completely absent or underdeveloped in *Caucasirella* gen. nov. (Sidorov 2015; Marin and Palatov 2021b). Another key distinction is the distal margin of the telson, which is shallowly notched in *L. hyrcana* (see Sidorov 2015: Fig. 8G, L), whereas distinctly notched in *Caucasirella* gen. nov. (Sidorov 2015; Marin and Palatov 2021b), even in juveniles (see Fig. 13f).

Neoteny is a common feature among crangonyctid amphipods in general and particularly within the “*Synurella*” clade (Palatov and Marin 2020; Marin et al. 2023; Copilaș-Ciocianu et al. 2024). In addition to the widely accepted classical hypothesis that neotenic development is driven by extreme habitat conditions and serves to conserve body resources, we hypothesize that such morphological adaptations promote speciation by expanding organisms’ ability to inhabit specific microhabitats, such as fissures, narrow pores, and tectonic faults (e.g., Marin et al. 2023). Within the “*Palearcticarellus* + *Eosynurella*” clade, all representatives of *Palearcticarellus* possess simple dactyli on their ambulatory pereopods (Martynov 1930; Kulkina 1992; Sidorov et al. 2010; Palatov and Marin 2020; Marin et al. 2023), while adults of *Eosynurella* have additional spine-like setae along the inner margin (Derzhavin 1930; Martynov 1931; Karaman 1990), which are similar to those found in *Lyurella*. However, we suggest that ancestor characterized by simple dactyli lacking these additional spine-like setae, since it is simple in juveniles (see Fig. 13). In small-sized species of *Eosynurella* (possible juveniles or neotenic species), the dactyli are also simple (e.g., Sidorov et al. 2010; Copilaş -Ciocianu et al. 2024). In this regard, the taxonomic position of *Palearcticarellus hyperboreus* Sidorov & Copilaş-Ciocianu, 2024 (Copilaş-Ciocianu et al. 2024) need to be verified, since its phylogenetic position (Fig. 1), morphology and distribution (Fig. 14) argue that it may be a either a juvenile or neotenic species of the genus *Eosynurella*. Nevertheless, further studies are required to confirm this hypothesis and clarify the taxonomic status of the respective taxa. This also underscores the importance of describing juvenile morphology during early developmental stages for taxonomic research, as it can provide valuable insights into phylogenetic and evolutionary processes. Furthermore, juveniles of Crangonyctidae are often found in the marsupia of collected ♀♀, making them readily accessible for researchers. We encourage the scientific community to pay close attention to these findings, as they can contribute to a better understanding of the life cycles and ecological roles of these crustaceans.

## Supplementary Material

XML Treatment for Caucasirella

XML Treatment for Caucasirella fanagorica

XML Treatment for Caucasirella asheensis

XML Treatment for Caucasirella fontinalis

XML Treatment for Caucasirella shapsuhica

XML Treatment for Caucasirella lazareviensis

XML Treatment for Caucasirella mzymtiensis

## Additional information

### Artificial Intelligence (AI) use

The authors accept full responsibility for the content of the manuscript, including the disclosure of any use of AI.

No AI tools were used in the preparation of this manuscript.

### Author ORCIDs

I.N. Marin https://orcid.org/0000-0003-0552-8456

D.M. Palatov https://orcid.org/0000-0002-8826-9316

### Data availability

All of the data that support the findings of this study are available in the main text.
